# Benzoate mediates the simultaneous repression of anaerobic 4-methylbenzoate and succinate utilization in *Magnetospirillum* sp. strain pMbN1

**DOI:** 10.1186/s12866-014-0269-4

**Published:** 2014-10-27

**Authors:** Sven Lahme, Kathleen Trautwein, Annemieke Strijkstra, Marvin Dörries, Lars Wöhlbrand, Ralf Rabus

**Affiliations:** Institute for Chemistry and Biology of the Marine Environment (ICBM), Carl von Ossietzky University Oldenburg, Oldenburg, Germany; Max Planck Institute for Marine Microbiology, Bremen, Germany

**Keywords:** Anaerobic degradation, 4-Methylbenzoate, Aromatic compounds, Repression, Diauxie, Ternary substrate mixture, Physiology, Transcript analysis, Proteomics

## Abstract

**Background:**

At high concentrations of organic substrates, microbial utilization of preferred substrates (i.e., supporting fast growth) often results in diauxic growth with hierarchical substrate depletion. Unlike the carbon catabolite repression-mediated discriminative utilization of carbohydrates, the substrate preferences of non-carbohydrate-utilizing bacteria for environmentally relevant compound classes (e.g., aliphatic or aromatic acids) are rarely investigated. The denitrifying alphaproteobacterium *Magnetospirillum* sp. strain pMbN1 anaerobically degrades a wide variety of aliphatic and aromatic compounds and is unique for anaerobic degradation of 4-methylbenzoate. The latter proceeds via a distinct reaction sequence analogous to the central anaerobic benzoyl-CoA pathway to intermediates of central metabolism. Considering the presence of these two different anaerobic “aromatic ring degrading” pathways, substrate preferences of *Magnetospirillum* sp. strain pMbN1 were investigated. Anaerobic growth and substrate consumption were monitored in binary and ternary mixtures of 4-methylbenzoate, benzoate and succinate, in conjuction with time-resolved abundance profiling of selected transcripts and/or proteins related to substrate uptake and catabolism.

**Results:**

Diauxic growth with benzoate preference was observed for binary and ternary substrate mixtures containing 4-methylbenzoate and succinate (despite adaptation of *Magnetospirillum* sp. strain pMbN1 to one of the latter two substrates). On the contrary, 4-methylbenzoate and succinate were utilized simultaneously from a binary mixture, as well as after benzoate depletion from the ternary mixture. Apparently, simultaneous repression of 4-methylbenzoate and succinate utilization from the ternary substrate mixture resulted from (i) inhibition of 4-methylbenzoate uptake, and (ii) combined inhibition of succinate uptake (via the two transporters DctPQM and DctA) and succinate conversion to acetyl-CoA (via pyruvate dehydrogenase). The benzoate-mediated repression of C_4_-dicarboxylate utilization in *Magnetospirillum* sp. strain pMbN1 differs from that recently described for “*Aromatoleum aromaticum*” EbN1 (involving only DctPQM).

**Conclusions:**

The preferential or simultaneous utilization of benzoate and other aromatic acids from mixtures with aliphatic acids may represent a more common nutritional behavior among (anaerobic) degradation specialist than previously thought. Preference of *Magnetospirillum* sp. strain pMbN1 for benzoate from mixtures with 4-methylbenzoate, and thus temporal separation of the benzoyl-CoA (first) and 4-methylbenzoyl-CoA (second) pathway, may reflect a catabolic tuning towards metabolic efficiency and the markedly broader range of aromatic substrates feeding into the central anaerobic benzoyl-CoA pathway.

**Electronic supplementary material:**

The online version of this article (doi:10.1186/s12866-014-0269-4) contains supplementary material, which is available to authorized users.

## Background

Aromatic compounds are widely distributed and abundant constituents of natural organic matter [1], and support as energy-rich substrates the growth of heterotrophic bacteria. In the environment, aromatic compounds typically co-occur with various and often more easily degradable substrates (e.g., aliphatic acids, carbohydrates, amino acids). Despite the high diversity of potential carbon sources, their total concentration generally does not exceed the μM range. These low concentrations cause microorganisms to simultaneously utilize multiple different substrates [2,3]. However, higher (up to mM) substrate concentrations are expected to occur due to transient pulses (e.g., collapse of algal blooms, oil spills) or continuous release (e.g., petrochemical waste waters).

Laboratory experiments with batch cultures of microorganisms often show biphasic (diauxic) growth with hierarchical utilization of substrates. Firstly, the preferred substrate is exclusively and completely consumed during the first active growth phase. Then, utilization of the subordinate substrate starts following an intermediarily occuring diauxic lag phase [4]. The mechanisms underlying substrate utilization preferences were termed carbon catabolite repression (CCR) and have been studied intensively for carbohydrates in a limited number of bacteria, and involve different phosphotransferase systems (PTS) for sugar uptake [5,6]. The PTS-independent sugar uptake in *Pseudomonas* spp. [7] and the inability of many nutritional specialists (e.g., members of the betaproteobacterial “*Aromatoleum*”*/Azoarcus/Thauera* cluster) to utilize carbohydrates, implies the existence of control mechanisms that deviate from the known concepts established with well-studied *Escherichia coli* and *Bacillus subtilis*. In bacteria able to anaerobically degrade aromatic compounds, few studies have investigated substrate preferences thus far. Under nitrate-reducing conditions and growth at comparable rates, succinate-adapted *Azoarcus* sp. strain CIB preferred utilization of succinate from a binary mixture with benzoate [8], which is mediated by the regulatory protein AccR [9]. In contrast to *Azoarcus* sp. strain CIB, related “*Aromatoleum aromaticum*” EbN1 preferred benzoate over succinate by repressing succinate uptake via the C_4_-dicarboxylate TRAP transporter DctPQM [10].

The denitrifying alphaproteobacterium *Magnetospirillum* sp. strain pMbN1 is the only known isolate utilizing 4-methylbenzoate under anoxic conditions [11]. 4-Methylbenzoate and benzoate are degraded anaerobically via two distinct, yet analogous pathways [12] that involve: (i) substrate-CoA ligation, (ii) reductive dearomatization of (4-methyl)benzoyl-CoA by dedicated 4-methylbenzoyl-CoA (MbrBCAD) or benzoyl-CoA (BcrCBAD) reductase, and (iii) a series of β-oxidation-like reactions to yield 3-hydroxy-5-methylpimelyl-CoA (4-methylbenzoate-derived) or 3-hydroxypimelyl-CoA (benzoate-derived). Further degradation of the latter two intermediates to acetyl-CoA then proceeds differently either via reactions analogous to the leucine/isovalerate pathway or by β-oxidation, respectively [12]. In the environment, 4-methylbenzoate and benzoate were reported to co-occur in wastewaters from terephthalic acid production plants at concentrations ranging from 0.4 to 4.1 mM [13,14].

This study combines physiology with differential transcript and protein analyses to investigate the substrate preferences of *Magnetospirillum* sp. strain pMbN1 during anaerobic growth with binary and ternary mixtures of 4-methylbenzoate, benzoate and succinate.

## Results

### Substrate utilization preferences of *Magnetospirillum* sp. strain pMbN1

**Figure 1:**
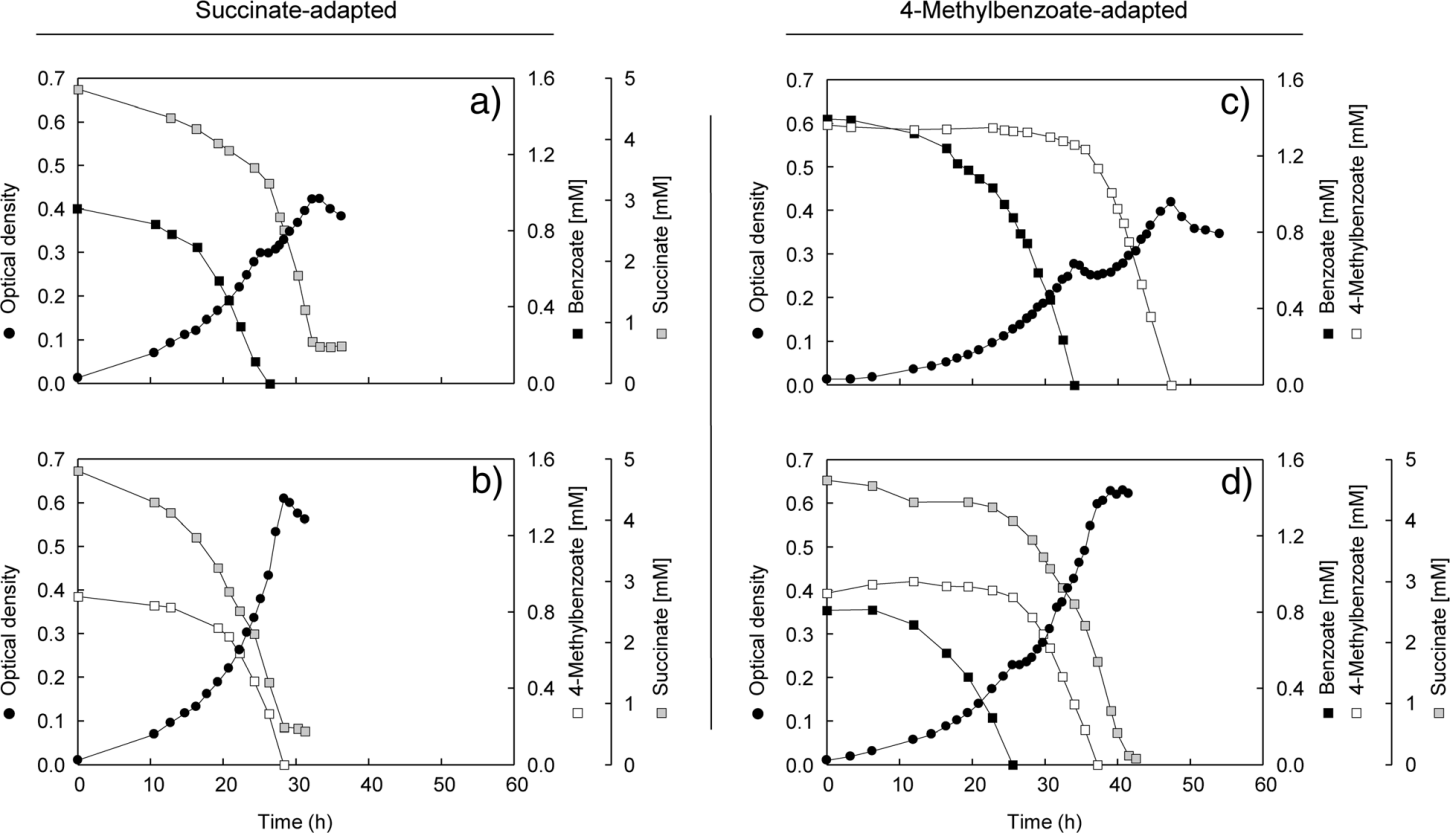
**Anaerobic growth of** ***Magnetospirillum*** **sp. strain pMbN1 with binary and ternary substrate mixtures of 4-methylbenzoate, benzoate and succinate.** Growth behavior and substrate utilization profiles of *Magnetospirillum* sp. strain pMbN1 adapted to succinate **(a,b)** or 4-methylbenzoate **(c,d)**.

**Binary substrate mixtures.** Succinate-adapted cells of *Magnetospirillum* sp. strain pMbN1 growing with a mixture of succinate and benzoate displayed diauxic growth (Figure 1a). In the first active growth phase (phase 1), the cells preferentially and completely consumed benzoate, while already part of the supplied succinate (26%) was slowly depleted from the medium. Following a 2.1 h long diauxic lag phase (phase 2), succinate was rapidly utilized in the second active growth phase (phase 3). In contrast to benzoate, other tested co-substrates (4-methylbenzoate [Figure 1b], phenylacetate or acetate) were co-utilized with succinate (monophasic growth) or succinate was preferred (4-hydroxybenzoate, diauxic growth) (Table 1; Additional file 1: Figure S1). Benzoate was also preferentially utilized (diauxic growth) from binary mixtures containing the adaptation substrates fumarate, L-malate, oxaloacetate or pyruvate (Table 1; Additional file 1: Figures S2 and S3). In analogy to succinate, also here the adaptation substrates were already partially and slowly depleted during benzoate utilization with the duration of diauxic lag phases ranging from 1.0 to 2.6 h. Only in combination with acetate as adaptation substrate, monophasic growth with co-utilization of benzoate was observed. In contrast, when supplying 4-methylbenzoate as co-substrate, diauxic growth with exclusive and preferential consumption of acetate was observed (3.5 h long diauxic lag phase), whereas pyruvate and 4-methylbenzoate were co-utilized (monophasic growth) (Table 1; Additional file 1: Figure S3). Using cells adapted to 4-methylbenzoate, both tested aromatic co-substrates (benzoate [Figure 1c], phenylacetate) were exclusively and completely utilized during phase 1, followed by a 4–5 h long diauxic lag phase and utilization of 4-methylbenzoate only during phase 3 (Table 1; Additional file 1: Figure S1).

**Table 1:** **Anaerobic growth of** ***Magnetospirillum*** **sp. strain pMbN1 with binary mixtures of aliphatic and aromatic acids**

		**Binary mixture of adaptation and co-substrate**				
		**Phase 1**		**Phase 2**	**Phase 3**	
**Adaptation substrate**	**Co-substrate**	**OD**_**max**_	**μ**_**max**_ **(h** ^**−1**^ **)**	**Duration of diauxic lag phase (h)**	**OD**_**max**_	**μ**_**max**_ **(h** ^**−1**^ **)**
*Cells adapted to succinate*						
Succinate (5 mM)	**Benzoate (1 mM)**	0.30	0.19	2.1 ± 0.1	0.42	0.28
Succinate (5 mM)	4-Methylbenzoate (1 mM)	0.61	0.24	None	None	None
**Succinate (5 mM)**	4-Hydroxybenzoate (1 mM)	0.43	0.27	3.3 ± 0.1	0.51	0.50
Succinate (5 mM)	Phenylacetate (1 mM)	0.66	0.25	None	None	None
Succinate (5 mM)	Acetate (8 mM)	0.53	0.23	None	None	None
*Cells adapted to 4-methylbenzoate*						
4-Methylbenzoate (1.5 mM)	**Benzoate (1.5 mM)**	0.27	0.29	4.0 ± 0.0	0.41	0.29
4-Methylbenzoate (1.5 mM)	**Phenylacetate (1.5 mM)**	0.30	0.29	5.0 ± 0.0	0.45	0.28
*Cells adapted to other aliphatic acids*						
Acetate (8 mM)	Benzoate (1 mM)	0.38	0.18	None	None	None
**Acetate (8 mM)**	4-Methylbenzoate (1 mM)	0.25	0.16	3.5 ± 0.0	0.32	0.24
Pyruvate (6 mM)	**Benzoate (1 mM)**	0.30	0.17	1.3 ± 0.3	0.54	0.37
Pyruvate (6 mM)	4-Methylbenzoate (1 mM)	0.54	0.22	None	None	None
Fumarate (5 mM)	**Benzoate (1 mM)**	0.30	0.17	2.3 ± 0.0	0.43	0.29
L-Malate (5 mM)	**Benzoate (1 mM)**	0.31	0.44	2.6 ± 0.1	0.49	0.28
Oxaloacetate (6 mM)	**Benzoate (1 mM)**	0.31	0.19	1.0 ± 0.0	0.42	0.34
*Cells adapted to other aromatic acids*						
4-Hydroxybenzoate (1 mM)	**Succinate (5 mM)**	0.43	0.32	4.3 ± 0.0	0.49	0.50

Preferentially utilized substrates are highlighted in boldface. Applied substrate concentrations are indicated in parentheses. Values for maximal optical density (OD_max_) and maximum specific growth rates (μ_max_) are based on three replicate cultures with standard deviations of below 5%. μ_max_ was calculated from the slope of the active growth phase (*m*) according to μ_max_ = *m* × 1/ΔOD. In the case of diauxic growth, phase 1 corresponds to the first active growth phase. In the case of monophasic growth, phase 1 corresponds to the only observed active growth phase. If present, phase 3 corresponds to a second active growth phase, with phase 2 representing the diauxic lag phase between phases 1 and 3 (Figure 1). For further details see Additional file 1: Figures S1–S3.

**Ternary substrate mixture.** Cells adapted to 4-methylbenzoate were shifted to a mixture of 4-methylbenzoate, benzoate and succinate, resulting in diauxic growth with preferential and complete utilization of benzoate during phase 1 (Table 2, Figure 1d). Again, this was accompanied by partial (15%) depletion of the supplied succinate. Following a short (1.8 h) diauxic lag phase, 4-methylbenzoate and the bulk of succinate were co-utilized in phase 3. The same growth behavior and substrate preference was observed, if succinate-adapted cells were used as inoculum (data not shown). A notable observation is the unaltered utilization hierarchy with the ternary mixture, i.e. benzoate over succinate and 4-methylbenzoate, mirroring the preferences observed with respective binary mixtures (Figure 1a–c).

**Table 2:** **Anaerobic growth of** ***Magnetospirillum*** **sp. strain pMbN1 with a ternary mixture of 4-methylbenzoate, benzoate and succinate**

			**Ternary mixture of adaptation and co-substrates**				
			**Phase 1**		**Phase 2**	**Phase 3**	
**Adaptation substrate**	**Co-substrate 1**	**Co-substrate 2**	**OD**_**max**_	**μ**_**max**_ **(h** ^**−1**^ **)**	**Duration of diauxic lag phase (h)**	**OD**_**max**_	**μ**_**max**_ **(h** ^**−1**^ **)**
4-Methylbenzoate (1 mM)	**Benzoate (1 mM)**	Succinate (5 mM)	0.23	0.16	1.8 ± 0.1	0.62	0.31

For details see description to Table 1.

### Transcript and protein dynamics (ternary substrate mixture)

**Figure 2:**
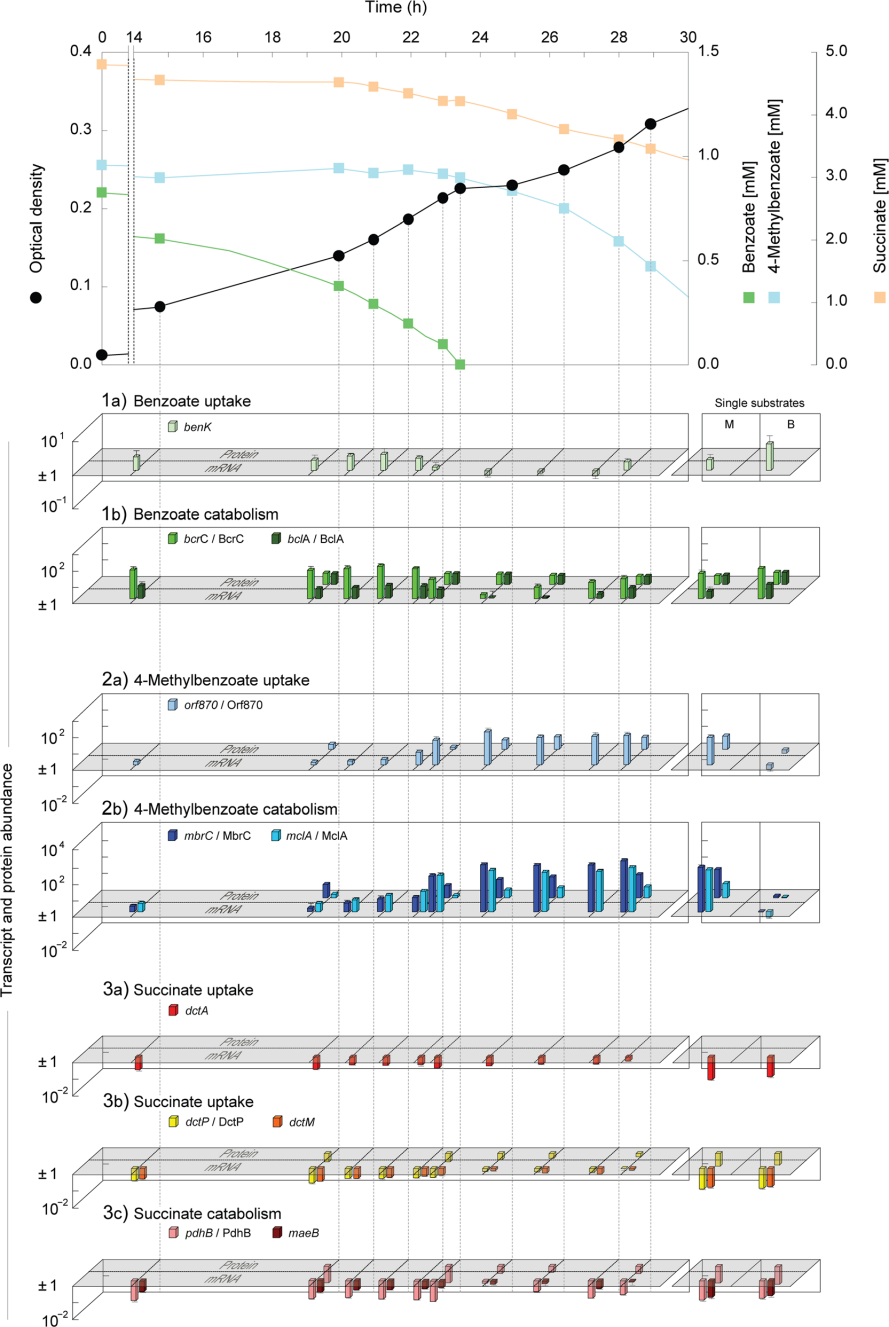
**Time-resolved profiles of selected transcripts and corresponding proteins (if identified by 2D DIGE) related to uptake and catabolism of benzoate (1ab), 4-methylbenzoate (2ab) and succinate (3abc).** 4-Methylbenzoate-adapted cells of *Magnetospirillum* sp. strain pMbN1 were shifted to a ternary substrate mixture (see Figure 1d) of 4-methylbenzoate (adaptation substrate), benzoate (co-substrate) and succinate (co-substrate). Abbreviations for experiments with single substrates: M, 4-methylbenzoate; B, benzoate. The fold change in protein and ratio of transcript abundance were determined by comparison to succinate-adapted cells as reference. See legend to Figure 3 for name and predicted functions of selected transcripts/proteins. For detailed information see Additional file 1: Tables S1 and S2.

**Figure 3:**
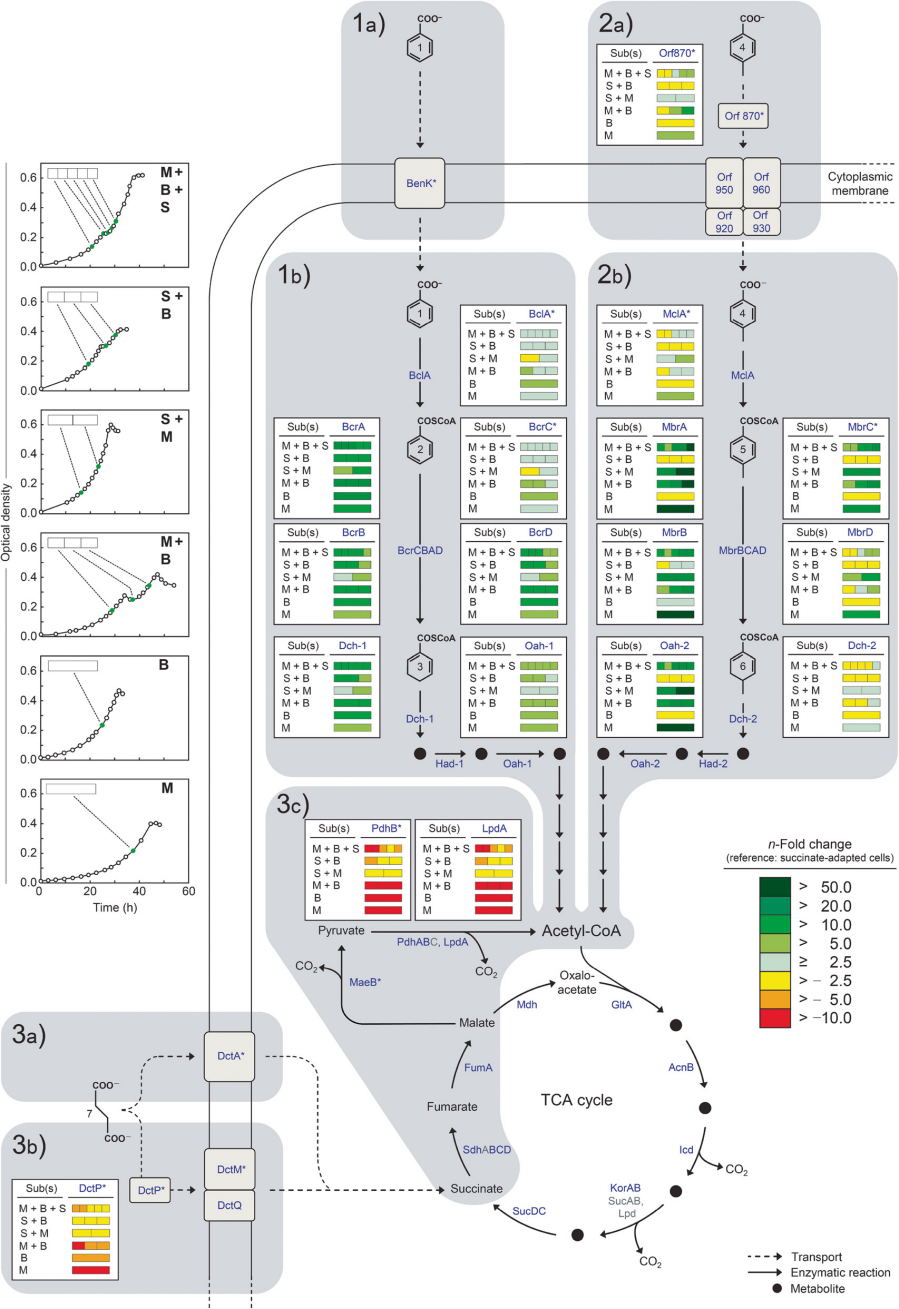
**Fold change in the abundance of proteins related to uptake and catabolism of benzoate (1ab), 4-methylbenzoate (2ab) and succinate (3abc) during anaerobic growth of** ***Magnetospirillum*** **sp. strain pMbN1 with substrate mixtures or single substrates (M, 4-methylbenzoate; B, benzoate; S, succinate; the adaptation substrate is listed first in each case).** Sampling points for proteomic analyses are marked in green in the growth curves displayed on the left panel. Identified proteins are marked in blue. For further details (including abundance profiles of Had-1 and Had-2) see Additional file 1: Table S1. *, Transcript profiles of selected genes during growth with the ternary substrate mixture are shown in Figure 2. Protein names (in alphabetical order): (AcnB) aconitase 2, (BclA) benzoate-CoA ligase, (BcrCBAD) benzoyl-CoA reductase, (BenK) benzoate/H^+^ symporter, (Dch-1) cyclohex-1,5-diene-1-carbonyl-CoA hydratase, (Dch-2) 4-methylcyclohex-1,5-diene-1-carbonyl-CoA hydratase, (DctA) C_4_-dicarboxylate/Na^+^ symporter, (DctPQM) TRAP-type C_4_-dicarboxylate uptake transporter, (FumA) fumarate hydratase, (GltA) citrate synthase, (Had-1) 6-hydroxycyclohex-1-ene-1-carbonyl-CoA dehydrogenase, (Had-2) 6-hydroxy-4-methylcyclohex-1-ene-1-carbonyl-CoA dehydrogenase, (Icd) isocitrate dehydrogenase, (KorAB) 2-oxoglutarate:ferredoxin oxidoreductase, (MaeB) NADP^+^-dependent malic enzyme, (MbrCBAD) 4-methylbenzoyl-CoA reductase, (MclA) 4-methylbenzoate-CoA ligase, (Mdh) malate dehydrogenase, (Oah-1) 6-oxocyclohex-1-ene-1-carbonyl-CoA hydrolase, (Oah-2) 4-methyl-6-oxocyclohex-1-ene-1-carbonyl-CoA hydrolase, (Orf870–960) predicted ABC-type 4-methylbenzoate uptake transporter, (PdhABC, LpdA) pyruvate dehydrogenase complex, (SdhABCD) succinate dehydrogenase, (SucAB, Lpd) 2-oxoglutarate dehydrogenase complex, (SucDC) succinyl-CoA ligase. Compound names: 1, benzoate; 2, benzoyl-CoA; 3, cyclohex-1.5-diene-carbonyl-CoA; 4, 4-methylbenzoate; 5, 4-methylbenzoyl-CoA; 6, 4-methylcyclohex-1.5-diene-1-carbonyl-CoA; 7, succinate.

Time-resolved abundance profiles of transcripts (11 different across 10 time points) and proteins (soluble and membrane, 5 time points) related to the uptake and catabolism of 4-methylbenzoate, benzoate and succinate were determined during anaerobic diauxic growth with the ternary substrate mixture, and related to that of succinate-grown cells as reference (Figures 2 and 3). For comparison, the same transcripts/proteins were profiled accordingly at lower time resolution (1–3 time points) for respective binary substrate mixtures (Figure 1a–c) and single substrates.

**Benzoate uptake and catabolism (Figure** **2****:1a and b).** Benzoate utilization during phase 1 coincided with the largest abundance increase (up to 24.3-fold) for the protein constituents of the anaerobic benzoyl-CoA pathway (BclA, BcrCBAD, Dch-1, Had-1, and Oah-1), which then constantly decreased during phases 2 and 3 (Additional file 1: Tables S1 and S2). Immediately (within 30 min) after complete depletion of benzoate from the medium, the transcript level of the C-subunit of benzoyl-CoA reductase (*bcrC*) decreased markedly (from 60- to 13-fold) and reached its minimum (2-fold) at the end of the diauxic lag phase. Transcripts of benzoate-CoA ligase (*bclA*) behaved similarly, but the overall abundance change was far less pronounced (up to 5-fold) (Additional file 1: Table S2).

Benzoate uptake in *Magnetospirillum* sp. strain pMbN1 is predicted to proceed via a benzoate/H^+^ symporter homologous to BenK [15]. In cells of *Magnetospirillum* sp. strain pMbN1, BenK was detected during growth with benzoate or 4-methylbenzoate, but not with succinate. In contrast to the catabolic constituents of the anaerobic benzoyl-CoA pathway, transcript and protein levels of BenK changed only slightly across phases 1–3 (Additional file 1: Tables S2 and S3).

**4-Methylbenzoate uptake and catabolism (Figure** **2****: 2a and b).** Despite non-utilization of 4-methylbenzoate during phase 1 of diauxic growth, four proteins of the anaerobic 4-methylbenzoyl-CoA pathway (MclA, **MbrBCA**D, Dch-2, Had-2, and **Oah-2**) displayed increased abundances (proteins marked in bold: 6.5- to 11.6-fold). At the end of the diauxic lag phase, these proteins markedly increased in abundance, reaching their highest levels during maximal 4-methylbenzoate utilization in phase 3 (up to 60-fold for MbrC). Transcript levels of predicted 4-methylbenzoate-CoA ligase (*mclA*) and of the C-subunit of 4-methylbenzoyl-CoA reductase (*mbrC*) were initially low (up to 3-fold), but started to increase at benzoate concentrations <0.3 mM, which was followed by a strong increase upon benzoate depletion (up to 139-fold), and likewise reaching maximal levels (up to 1046-fold) in phase 3 (Additional file 1: Table S2). Incongruent transcript (low) and protein (medium) levels of MbrC during phase 1 suggest that the highly abundant proteins of 4-methylbenzoate degradation [12] might only be slowly degraded.

At present, the proteomic dataset suggests uptake of 4-methylbenzoate by an ABC transporter, most subunits of which (Orf870, 920, 930, 950, 960) were present in cells grown with 4-methylbenzoate, but absent in cells grown with succinate or benzoate (Additional file 1: Table S3). The transcript level of *orf870* immediately increased at the end of phase 1 (from 6- to 34-fold within 30 min after benzoate depletion), peaked at the end of the diauxic lag phase (108-fold), and remained at high levels during phase 3 (~50-fold) (Additional file 1: Table S2). The protein level of Orf870 changed accordingly and the four membrane-associated components (Orf920–960) of the ABC transporter were identified only in the membrane protein fraction of cells harvested during phases 2 and 3 (Additional file 1: Tables S1 and S3).

**Succinate uptake and catabolism (Figure** **2****:3a, b and c).** Among all identified proteins, the β-subunit (PdhB) of the E1 and the E3 (LpdA) component of pyruvate dehydrogenase (PdhABC, LpdA) displayed the largest decreases in abundance (up to −10.3-fold in phase 1). Notably, during bulk succinate utilization in phase 3, both proteins attained similarly high levels as observed in the succinate-grown reference (Additional file 1: Table S1). The changes in transcript levels of *pdhB* essentially correlated with changes in the PdhB protein level. In addition to pyruvate dehydrogenase, metabolism of succinate to pyruvate is suggested to also involve NADP^+^-dependent malic enzyme (MaeB), which was not detected by 2D DIGE. Only at benzoate concentrations >0.4 mM, the transcript level of *maeB* was slightly (5-fold) reduced (Additional file 1: Table S2). The abundance profiles of MaeB and pyruvate dehydrogenase agree with their dispensable function during benzoate or 4-methylbenzoate catabolism (i.e., both pathways converge at acetyl-CoA). Correspondingly, minimal transcript/protein abundances of these two enzymes were observed when the two aromatic substrates were utilized (supplied as single substrates or mixture) (Additional file 1: Tables S1 and S2).

Based on the proteomic dataset, uptake of succinate in *Magnetospirillum* sp. strain pMbN1 is suggested to involve two different transporters. Firstly, a C_4_-dicarboxylate TRAP transporter, displaying high amino acid sequence identities (69, 52 and 77%, respectively) to characterized DctPQM from *Rhodobacter capsulatus* [16-18], and secondly, the C_4_-dicarboxylate/cation symporter DctA with 63% amino acid sequence identity to characterized DctA from *Sinorhizobium meliloti* [19]. 2D DIGE captured only the periplasmic C_4_-dicarboxylate-binding protein DctP of the TRAP transporter, which displayed decreased abundance (−3.4-fold) during benzoate utilization in phase 1, and increased continuously upon benzoate depletion during phases 2 and 3 (Additional file 1: Table S1). DctQM were both identified in the membrane protein-enriched fraction of succinate-grown cells, whereas only one of the two subunits was detected in cells grown with benzoate, or across phases 1–3. Symporter DctA was identified in succinate-grown cells and across phases 1–3, but not in cells grown with benzoate or 4-methylbenzoate as single substrate (Additional file 1: Table S3). Transcript levels of *dctP*, *dctM* and *dctA* were similar across phases 1–3, the extent and dynamics of which were comparable to that of the *maeB* transcript (see above). Minimal transcript levels of *dctP*, *dctM* and *dctA* were observed during growth with benzoate or 4-methylbenzoate as single substrate (Additional file 1: Table S2). These transcript profiles are in accord with the known C_4_-dicarboxylate-dependent activation of *dctPQM* and *dctA* expression by dedicated C_4_-dicarboxylate-responsive two-component sensory/regulatory systems as known from *R. capsulatus* (DctSR) and rhizobia (DctBD) [20,21]. Homologues of those are also encoded in the genome of *Magnetospirillum* sp. strain pMbN1 (Additional file 1: Table S4).

**Ternary*versus*binary substrate mixtures (Figure****3****).** Protein profiles of cells grown with selected binary mixtures (succinate with benzoate or 4-methylbenzoate; 4-methylbenzoate with benzoate) were compared to those from cultures grown with the ternary substrate mixture. Overall, the dynamics and extent of protein abundance changes matched those observed with the ternary substrate mixture. However, differences were observed for some proteins (Additional file 1: Table S1): (i) the protein constituents of the anaerobic benzoyl-CoA pathway were generally higher in abundance, if 4-methylbenzoate was additonally present. (ii) In the absence of benzoate, DctP and PdhB levels were unaffected by 4-methylbenzoate, agreeing with co-utilization of succinate and 4-methylbenzoate (Figure 1b). However, if provided together, 4-methylbenzoate and benzoate had an additive negative effect on these two proteins, which could contribute to the observed decreased succinate depletion during phase 1 with the ternary substrate mixture (Figure 1a *versus* d). It should be noted, however, that the observed differences might be related to using either succinate or 4-methylbenzoate as adaptation substrate in the compared experiments. (iii) The abundance change of DctP and PdhB was incongruent in the presence of succinate (ternary substrate mixture *versus* binary mixture of benzoate and 4-methylbenzoate). I.e., DctP levels increased from −5.7- to −3.4-fold in phase 1, while PdhB levels remained minimal at −9.9-fold, thus indicating that unlike DctP, repression of PdhB is unaffected by the co-substrate succinate.

## Discussion

### Benzoate is preferred by *Magnetospirillum* sp. strain pMbN1

*Magnetospirillum* sp. strain pMbN1 is currently the only described isolate capable of anaerobically degrading 4-methylbenzoate [11]. Anaerobic degradation of 4-methylbenzoate and benzoate proceeds via two analogous pathways each requiring a distinct set of enzymes [12]. Despite adaptation to 4-methylbenzoate and thereby “transferred” initial presence of catabolic proteins for 4-methylbenzoate degradation, *Magnetospirillum* sp. strain pMbN1 preferred utilization of benzoate from mixtures with 4-methylbenzoate (Figure 1c and d). This preference cannot be attributed to markedly different growth rates (i.e., 0.15–0.21 h^−1^ for benzoate, and 0.13–0.19 h^−1^ for 4-methylbenzoate) (Table 1). However, lower growth yields (8.5 g *versus* 9.5 g (mol C)^−1^ for benzoate) despite the higher free energies (Δ*G*^0′^) obtained from complete oxidation of 4-methylbenzoate (−3583 kJ mol^−1^*versus −*2973 kJ mol^−1^ for benzoate) [11], indicate less efficient metabolism with 4-methylbenzoate. In addition, the range of substrates feeding into the 4-methylbenzoyl-CoA degradation pathway is to our present knowledge limited to 4-methylbenzoate, whereas from the aromatic substrates of *Magnetospirillum* sp. strain pMbN1 [11], at least 11 (e.g., phenylacetate) are known from other denitrifying bacteria to be channeled into the central anaerobic benzoyl-CoA pathway (e.g., [22,23]). Indeed, also phenylacetate repressed 4-methylbenzoate utilization in *Magnetospirillum* sp. strain pMbN1 (Table 1). One may speculate that benzoate preference of *Magnetospirillum* sp. strain pMbN1 reflects the higher probability to encounter substrates of the anaerobic benzoyl-CoA pathway in the environment. In *Thauera aromatica* AR-1, benzoate repressed anaerobic 3,5-dihydroxybenzoate degradation, which is apparently degraded via a catabolic route distinct from the anaerobic benzoyl-CoA pathway [24,25]. It seems thus likely that the anaerobic benzoyl-CoA pathway might generally be preferred over more specialized (i.e., less central) aromatic degradation pathways.

Aerobic and anaerobic aromatic compound degrading bacteria in most reported cases prefer succinate over aromatic acids [8,26,27]. The opposite was described recently for the first time in the denitrifying betaproteobacterium “*A. aromaticum*” EbN1, prefering benzoate despite adaptation to anaerobic growth with succinate [10]. In this study, the same unusual preference was observed in the alphaproteobacterium *Magnetospirillum* sp. strain pMbN1, which is phylogenetically distinct from “*A. aromaticum*” EbN1. In addition to succinate, growth behavior and substrate preferences of both strains were similar with mixtures of benzoate and the adaptation substrates fumarate or malate (diauxic growth with benzoate preference in both cases) and acetate (monophasic growth with co-utilization of benzoate), but differed for oxaloacetate and pyruvate. While “*A. aromaticum*” EbN1 grew monophasically with one of the latter and benzoate, diauxic growth with benzoate preference was observed in *Magnetospirillum* sp. strain pMbN1. Substrate preferences in both strains could not be attributed to differences in growth rate (aliphatic substrates allowed overall 17 ± 4% faster growth) (Additional file 1: Table S5) [Table 1 in reference 10] and complexity of degradation pathways. Taken together, *Magnetospirillum* sp. strain pMbN1 and “*A. aromaticum*” EbN1 seem to be highly specialized on utilization of aromatic acids (“energy-rich”) under anoxic conditions, since aliphatic substrates (“energy-poor”) were first and exclusively utilized only from two (out of 23) tested binary substrate mixtures.

### Benzoate mediates repression of 4-methylbenzoate uptake

4-Methylbenzoate-specific detection of a putative ABC transporter for 4-methylbenzoate uptake (Orf870–960) and the strong increase in transcript levels of its periplasmic solute-binding protein (*orf870*) upon benzoate depletion (Figure 2), suggest benzoate to mediate repression of 4-methylbenzoate uptake. This is supported by non-utilization of 4-methylbenzoate during phase 1, despite presence of some catabolic proteins (MbrBCA, Oah-2) (Figure 3). Expression of genes for anaerobic catabolism of aromatic acids is typically controlled by regulatory proteins recognizing corresponding aryl-CoA esters (e.g., [28-31]). Inhibition of 4-methylbenzoate uptake would prevent its intracellular presence and thus generation of 4-methylbenzoyl-CoA as putative inductor for expression of genes for 4-methylbenzoate catabolism. This is evident, e.g., from observed minimal transcript levels of *mclA*, encoding 4-methylbenzoate-CoA ligase, and *mbrC* during phase 1. It is known that benzoate-CoA ligases of *Azoarcus* sp. strain CIB [8], *T. aromatica* K172 [32] and *Magnetospirillum* sp. strain TS-6 [33] also convert 4-fluorobenzoate at high rates (4-methylbenzoate not tested), and 3-methylbenzoate-CoA ligase (MbdA) of *Azoarcus* sp. strain CIB exhibits the same activity with 3-methylbenzoate and benzoate [34]. The high (50%) amino acid sequence identities of benzoate-CoA ligase (BclA) of *Magnetospirillum* sp. strain pMbN1 and MbdA of *Azoarcus* sp. strain CIB may suggest BclA of *Magnetospirillum* sp. strain pMbN1 to possibly also accept 4-methylbenzoate as substrate. In this case, excluding 4-methylbenzoate from entering the cell would most effectively prevent induction of the 4-methylbenzoyl-CoA pathway.

### Benzoate mediates a dual repression of succinate uptake and utilization

In case of “*A. aromaticum*” EbN1, it was assumed that benzoate mediates complete repression of succinate uptake by negatively controlling DctSR-dependent activation of *dctPQM* expression [10]. Despite the similar diauxic growth behavior and presence of homologous DctPQM/DctSR (49–79% amino acid sequence identities), *Magnetospirillum* sp. strain pMbN1 partially depleted succinate already during benzoate utilization. This may be due to presence of an additional transporter for C_4_-dicarboxylate uptake (DctA) together with a cognate C_4_-dicarboxylate-responsive two-component sensory/regulatory system (DctBD) in *Magnetospirillum* sp. strain pMbN1. Simultaneous employment of DctA and DctPQM in succinate uptake has been reported for *Pseudomonas aeruginosa* PAO1, where DctBD (*dctSR* is not encoded in strain PAO1) controls the coordinate expression of *dctA* and *dctPQM* [35]. In *Magnetospirillum* sp. strain pMbN1, succinate-specific detection (transcript/protein) of DctA and DctPQM indeed suggests participation of both transporters in succinate uptake. The coding genes are apparently coordinately expressed, considering the similar dynamics in their transcript levels across phases 1–3 (Figures 2 and 3). The only slightly reduced levels of C_4_-dicarboxylate transporter transcripts during phase 1, suggest that benzoate does not have a similarly strong negative effect on DctSR- and/or DctBD-dependent transcriptional activation of *dctPQM*/*dctA* expression in *Magnetospirillum* sp. strain pMbN1, compared to “*A. aromaticum*” EbN1. Together with the partial depletion of succinate already during phase 1, repression of succinate uptake cannot be the only determinant for the observed diauxie in *Magnetospirillum* sp. strain pMbN1.

Succinate catabolism involves reactions of the TCA cycle, as well as malic enzyme (MaeB) and pyruvate dehydrogenase (PdhABC, LpdA) to generate acetyl-CoA. Reduced transcript/protein levels of the latter two enzymes in *Magnetospirillum* sp. strain pMbN1 during benzoate utilization in phase 1 (Figure 2), suggest benzoate to also mediate inhibition of succinate conversion to acetyl-CoA. The abundance decrease of the *pdhB* transcript, which was most pronounced and prolonged until complete benzoate depletion (Figure 2), indicates that benzoate could mediate transcriptional repression of pyruvate dehydrogenase as main target for inhibition of succinate catabolism. Since expression of pyruvate dehydrogenase genes requires intracellular pyruvate as inductor in *S. meliloti* [36] and *E. coli* [37], one may speculate that due to impeded succinate uptake together with reduced MaeB-dependent pyruvate generation, intracellular pyruvate levels are insufficient for activation of pyruvate dehydrogenase gene expression. The partial depletion (~1.5 mM) of pyruvate during preferred benzoate utilization (Additional file 1: Figure S3) could suggest a more direct involvement of benzoate in repressing the expression of pyruvate dehydrogenase genes. Apparently, also here full expression of the pyruvate dehydrogenase genes as basis for maximal pyruvate consumption could only be achieved upon complete depletion of benzoate.

Uptake of succinate, L-malate and fumarate in *Magnetospirillum* sp. strain pMbN1 most likely proceeds via DctPQM and DctA, whereas these transporters are generally not involved in import of pyruvate, oxaloacetate or acetate [16,38-40]. With the exception of acetate, pyruvate dehydrogenase is essential for intracellular conversion of these five aliphatic acids to acetyl-CoA. Co-utilization of acetate from a mixture with benzoate thus agrees with the importance of pyruvate dehydrogenase in achieving preferential benzoate utilization in *Magnetospirillum* sp. strain pMbN1.

### Possible regulatory mechanisms for simultaneous repression of 4-methylbenzoate and succinate utilization

At present we can only speculate about the mechanism(s) underlying benzoate-mediated repression of 4-methylbenzoate and succinate utilization in *Magnetospirillum* sp. strain pMbN1. In case of 4-methylbenzoate, a possible regulatory mechanism could involve the predicted TetR-type transcriptional regulator Orf880 (for gene position in relation to 4-methylbenzoate uptake and catabolic genes see reference [12]) and benzoyl-CoA as the common intermediate of anaerobic benzoate and phenylacetate degradation. One may speculate that in analogy to the TetR-type repressor DesT (inductor palmitoyl-CoA relieves repression, co-repressor oleoyl-CoA restores repression) [41], benzoyl-CoA may act as co-repressor of Orf880, re-establishing transcriptional repression of genes for 4-methylbenzoate uptake and catabolism in cells that were originally adapted to 4-methylbenzoate (i.e., potential inductor 4-methylbenzoyl-CoA present).

On the contrary, shared intermediates (benzoyl-CoA and/or acetyl-CoA) occuring in the anaerobic degradation of benzoate, 4-methylbenzoate, acetate, phenylacetate or 4-hydroxybenzoate, suggest benzoate itself as inductor to mediate the dual repression of C_4_-dicarboxylate uptake and catabolism. One may speculate that benzoate interferes directly or indirectly with DctSR- and/or DctBD-mediated signal transduction, e.g. by modulating kinase activities [42-44]. In addition, benzoate may activate transcriptional repressor(s) [45-48], facilitate degradation of target transcripts [49,50], or negatively affect translation of transcripts at the sensory/regulatory, uptake and catabolic level [26,51,52].

## Conclusions

Next to “*A. aromaticum*” EbN1, the only distantly related *Magnetospirillum* sp. strain pMbN1 is the second reported pure culture displaying an unexpected diauxie (“reversed” carbon catabolite repression) with benzoate preference from mixtures with C_4_-dicarboxylates. Despite the similar growth behavior, apparently different molecular mechanisms govern the sequential utilization in both strains, adding to the so far largely unexplored regulatory potential within anaerobic aromatic compound-degrading bacteria. Preferential utilization of aromatic acids, or their co-utilization with aliphatic acids, seems to be more common among these degradation specialists than previously expected. Co-utilization even at the applied high (mM) concentrations of aromatic and aliphatic acids further suggests only weak or actually absent carbon catabolite control.

The unique nutritional feature of *Magnetospirillum* sp. strain pMbN1 is the capacity to anaerobically grow with 4-methylbenzoate, in addition to aromatic acids degraded via the widespread anaerobic benzoyl-CoA pathway. One may speculate that the observed temporal separation of benzoate and 4-methylbenzoate utilization, and thereby successive operation of the benzoyl-CoA and 4-methylbenzoyl-CoA pathways allow preventing metabolic inefficiencies, e.g. by unbalanced draining of shared co-factors or the competitive inhibition of analogous enzymes.

## Methods

### Media and cultivation

The denitrifying bacterium *Magnetospirillum* sp. strain pMbN1 was cultivated in anoxic mineral medium with 10 mM nitrate [53]. Media were prepared as described previously in butyl rubber sealed 500-ml (or 250-ml) flat glass bottles, containing 400 ml (or 200 ml) of medium under an anoxic N_2_:CO_2_ (90:10, vol/vol) atmosphere [53]. Substrates were added from sterile aqueous stock solutions. The calculated final concentration in the medium is listed in Tables 1 and 2. All chemicals used were of analytical grade.

### Growth experiments

The growth behavior of *Magnetospirillum* sp. strain pMbN1 was analyzed in the presence of various binary (Table 1) and one ternary (Table 2) substrate mixture(s). Cells were cultivated with each adaptation substrate for at least 5 passages before using 5% (vol/vol) of an actively growing culture to inoculate fresh medium for growth experiments (three replicate cultures per substrate mixture and two per single substrate). Over the time course of growth, samples of 3 ml were repeatedly removed from the cultures with sterile, N_2_-flushed syringes: 1 ml was used for monitoring the optical density (OD) at 660 nm (UV-mini 1202; Shimadzu, Duisburg, Germany) and 2 ml were immediately filtered (nitrocellulose, pore size 0.2 μm) and stored at −20°C for subsequent determination of substrate concentrations by high-performance liquid chromatography (HPLC). Negative controls lacked the inoculum and were treated in the same way.

### Harvesting for proteomic and targeted transcript analyses

To obtain sufficient cell material during anaerobic growth with substrate mixtures, *Magnetospirillum* sp. strain pMbN1 was cultivated in 5- or 10-liter Duran glass bottles (Ochs, Bovenden, Germany) filled with 4 or 8 liters of the anoxic medium described above. Bottles had three ports: one for gassing, one for inoculation or sampling, and one for repeated harvesting of cells during growth. Harvesting of cells for proteomic analyses was performed essentially as described by Champion et al. [54]. Resulting cell pellets were immediately frozen in liquid nitrogen and stored at −80°C.

Four different substrate mixtures were selected for proteomic analyses and cells were successively harvested from a large-scale culture: (i) during diauxic growth with succinate (adaptation substrate, 5 mM) and benzoate (co-substrate, 1 mM) in phase 1 (OD 0.17), phase 2 (OD 0.30), and phase 3 (OD 0.37) (Figure 1a), (ii) during monophasic growth with succinate (adaptation substrate, 5 mM) and 4-methylbenzoate (co-substrate, 1 mM) at two different time points (OD 0.14 and 0.30) (Figure 1b), (iii) during diauxic growth with 4-methylbenzoate (adaptation substrate, 1.5 mM) and benzoate (co-substrate; 1.5 mM) in phase 1 (OD 0.18), phase 2 (OD 0.24) and phase 3 (OD 0.33) (Figure 1c), and (iv) during diauxic growth with 4-methylbenzoate (adaptation substrate, 1 mM), benzoate (co-substrate, 1 mM) and succinate (co-substrate, 5 mM) in phase 1 (OD 0.14), at the transition into and out of phase 2 (both OD 0.23), and at the beginning (OD 0.25) and middle (OD 0.31) of phase 3 (Figure 1d). The latter culture was additionally sampled for targeted transcript analyses at 10 different time points during diauxic growth (Figure 2). At each time point, three times 5 or 10 ml portions of culture broth (depending on the OD) were directly collected with sterile glass pipettes. Subsamples (i.e., three technical replicates) were immediately mixed with two volumes of RNAprotect® Bacterial Reagent (Qiagen, Hilden, Germany), incubated for 5 min at room temperature and centrifuged (4,500 g, 30 min, 4°C). Pellets were resuspended in 0.5 ml RNAprotect® Bacterial Reagent and transferred into 2 ml microcentrifuge tubes prior to centrifugation (20,000 g, 5 min, 4°C). The supernatant was discarded and pellets were stored at −80°C.

For single substrates (succinate, 5 mM; benzoate, 2 mM; 4-methylbenzoate, 2 mM), three replicate cultures (400 ml medium in 500-ml flat glass bottles) each were harvested accordingly at an OD of ~0.20 for proteomic and targeted transcript analyses.

### High performance liquid chromatography (HPLC)

Substrate concentrations were determined with an UltiMate 3000 Rapid Separation LC system (ThermoScientific GmbH, Germering, Germany). Aliphatic organic acids were separated using a Eurokat H separation column (8 by 300 mm, 5 μm; Knauer, Berlin, Germany) that was temperature-controlled to 75°C. With 5 mM H_2_SO_4_ as the eluent and a flow rate of 0.8 ml min^−1^, aliphatic acids were detected at a wavelength of 210 nm. The retention times (with detection limits in parentheses) of UV-detected substrates were as follows: L-malate, 7.0 min (25 μM); oxaloacetate, 7.2 min (25 μM); pyruvate, 7.2 min (25 μM); succinate, 8.4 min (25 μM); fumarate, 9.3 min (5 μM); and acetate, 11.0 min (25 μM). Aromatic acids were analyzed on the same HPLC system equipped with a Dionex Acclaim 120 reversed-phase separation column (2.1 by 250 mm, 5 μm; ThermoScientific GmbH) that was temperature-controlled to 25°C. Separation was achieved with a non-linear gradient of acetonitrile (5–90%, vol/vol) as the eluent (pH adjusted to 2.8) at a flow rate of 0.5 ml min^−1^ as follows: 2 min at 5%, 5 to 14% in 1 min, 14 to 39% in 10.5 min, 39 to 90% in 3 min, and 3 min constant at 90%. Retention times (detection limit and compound-specific wavelength in parentheses) of UV-detected aromatic acids were as follows: 4-hydroxybenzoate, 7.5 min (1 μM, 260 nm); benzoate, 11.8 min (1 μM, 236 nm); phenylacetate, 12.2 min (1 μM, 195 nm); 4-methylbenzoate, 14.7 min (1 μM, 236 nm). Concentrations of succinate, benzoate, 4-methylbenzoate (shown in Figure 1) and phenylacetate were determined using a previously described HPLC method (see reference [10] for details).

### Soluble protein fraction: 2D DIGE and MALDI-TOF-MS/MS

Preparation of cell-free protein extracts and two-dimensional difference gel electrophoresis (2D DIGE) were performed as previously reported [55]. Cells were disrupted with the PlusOne sample grinding kit (GE Healthcare, Munich, Germany), and the protein concentration was determined as described by Bradford [56]. Isoelectric focusing (IEF) was performed using the IPGphor system (GE Healthcare) and commercial 24-cm immobilized pH gradient (IPG) strips with a non-linear pH gradient of 3 to 11 (GE Healthcare). The EttanDalt II system (GE Healthcare) was used for separation according to molecular mass in 12.5% acrylamide gels. Pre-electrophoretic labeling of proteins with different fluorescent dyes allowed co-separation of three samples in a single gel, representing the reference state (succinate, Cy5-labeled), the test state (variable, Cy3-labeled), and the pooled internal standard (Cy2-labeled). Individual test states (15 in total) were derived from cultures grown with four different substrate mixtures (see section on harvesting) or single substrates (benzoate or 4-methylbenzoate). The internal standard was composed of equal amounts of the reference and all 15 test states. 2D DIGE gels were scanned immediately after electrophoresis with a Typhoon 9400 scanner (GE Healthcare). Cropped gel images (four per test state) were analyzed with the DeCyder software (version 7.0; GE Healthcare). Applied parameters for spot detection and exclusion of non-proteinaceous spots were as described previously [22], resulting in 1608 ± 110 detected spots. Automatic matching of differentially abundant spots was manually controlled, which had to fulfill the following criteria: average ratio (*n*-fold change in protein abundance) of ≤ −2.5 or ≥2.5, analysis of variance (ANOVA) *P* value of <0.05, and *t* test value of <10^−4^, and matched in at least 45 of the 60 gels.

Protein spots with significant abundance changes were manually excised from preparative two-dimensional gel electrophoresis (2DE) gels (300 μg protein load) stained with colloidal Coomassie Brilliant Blue (cCBB) [57] and identified by mass spectrometry (MS). Tryptic digestion of excised 2DE-separated proteins was performed as previously described [58]. Peptide mass analysis and tandem MS was performed with an Ultraflextreme MALDI-TOF/TOF mass spectrometer (Bruker Daltonik GmbH, Bremen, Germany) operated as described recently [59]. Protein identification was performed with ProteinScape (version 3.1; Bruker Daltonik GmbH) on a Mascot server (version 2.3; Matrix Science Ltd, UK) based on a shotgun genome sequence dataset of *Magnetospirillum* sp. strain pMbN1 [12] translated into amino acid sequences as described recently [59].

### Membrane protein fraction: SDS-PAGE and nanoLC-ESI-MS/MS

Membrane protein-enriched fractions of cells grown with the ternary substrate mixture or with each of the three single substrates were prepared as described previously [60] and separated by SDS-PAGE (Bio-Rad, Munich, Germany) in 12.5% acrylamide gels (25 by 30 cm). Each sample lane selected for protein identification (Additional file 1: Figure S4) was subdivided into 14 gel slices, and each slice was further cut into pieces of ~1 mm^3^, prior to washing, reduction, alkylation and tryptic digestion [58]. Separation of peptides was performed with an Ultimate3000 nanoRSLC system (Thermo Scientific GmbH), online-coupled to an ion trap mass spectrometer (amazon ETD; Bruker Daltonik GmbH) as described previously [59]. Protein identification was performed as described above.

### Preparation of total RNA

All chemicals used during RNA preparation were of molecular biology grade. Total RNA was extracted from respective cell pellets within two weeks after harvest. (i) For single substrates, RNA was extracted from three biological replicates. (ii) For the ternary substrate mixture, RNA was extracted from three subsamples per time point (technical replicates). RNA extraction was essentially performed as previously described [61] using 60°C-hot saturated acidic phenol. The aqueous phase was then treated again with hot acidic phenol. After centrifugation, one volume of phenol/chloroform/isoamylalcohol (25:24:1) was added to the aqueous phase in 2 ml Phase Lock Gel™ tubes (5 Prime GmbH, Hamburg, Germany). Nucleic acids were subsequently precipitated with ice-cold ethanol (96% pure) during incubation at −80°C for 30 min. After centrifugation (20,000 g, 30 min, 4°C), the pellet was washed with 1 ml ice-cold ethanol (75%, vol/vol) and centrifuged again. The resulting pellet was dried and then resuspended in RNase-free water. Each RNA preparation was subjected to DNase I (RNase-free; Qiagen, Hilden, Germany) digestion. Removal of DNA was confirmed by PCR. RNA quality was controlled by the RNA 6000 Nano assay using an Agilent 2100 Bioanalyzer (Agilent Technologies, Böblingen, Germany). RNA concentration was determined using the Quant-iT™ Ribogreen® RNA assay kit (Life Technologies GmbH, Darmstadt, Germany). Total RNA was stored in aliquots at −80°C.

### Reverse transcription (RT) real-time PCR

Gene-specific primers (Additional file 1: Table S6) were designed for 11 target genes using the Lasergene software package (version 7.0.0; DNASTAR, Madison, WI, USA). Reverse transcription and real-time PCR detection were performed in a 20 μl one-tube reaction using the Brilliant III Ultra-Fast SYBR® Green QRT-PCR master mix (Agilent Technologies) and an IQ5 real-time PCR detection system (Bio-Rad). The one-tube RT real-time PCR reaction was performed as follows: one cycle of reverse transcription for 10 min at 50°C, followed by one cycle of PCR initiation for 3 min at 95°C, 40 cycles of 30 s denaturation at 95°C, 30 s annealing and extension at 60°C and real-time detection for 10 s between 82–87°C (Additional file 1: Table S6). Each reaction was performed with 5 ng of total RNA. The specificity of accumulated products was verified by melting curve analysis, ranging from 60–95°C in 0.5°C steps. Each RNA sample was analyzed by two (ternary substrate mixture) or four (single substrates) independent PCR reactions, corresponding to six (ternary substrate mixture) and 12 (single substrates) independent PCR reactions per analyzed time point.

The present physiological experiments are characterized by changes in (substrate-dependent) growth rates as well as intermediary lag phases. Such discrepant growth behaviors are known to affect the transcript abundance of reference “housekeeping” genes [62,63]. Therefore, we relinquished to use a reference gene for relative transcript quantification in the present study. The highly similar PCR efficiencies of each primer pair (standard deviation <0.08; Additional file 1: Table S7) allowed calculation of transcript abundance changes as ratio of the C_T_ values from the reference (succinate) and respective test state (i.e., 4-methylbenzoate, benzoate or ternary substrate mixture at 10 different time points) according to the following equation [64]:

$$ ratio = {E^{\Delta {C}_T}}^{\left( reference- test\right)} $$

Primer-specific efficiencies (E) of the PCR reaction for each primer pair were determined as previously reported [65].

### Nucleotide sequence accession numbers

Nucleotide sequences of genes discussed in this study were submitted to Genbank comprising accession numbers KF941494 to KF941542. A detailed list of proteins, accession numbers and manual annotation records is provided in Additional file 1: Table S4.

## Additional file

Additional file 1: Figure S1. Anaerobic growth and substrate utilization profiles of *Magnetospirillum* sp. strain pMbN1 with binary substrate mixtures of (A) succinate and 4-hydroxybenzoate, (B) succinate and phenylacetate, (C) succinate and acetate, and (D) 4-methylbenzoate and phenylacetate. **Figure S2.** Anaerobic growth and substrate utilization profiles of *Magnetospirillum* sp. strain pMbN1 with binary substrate mixtures of (A) fumarate and benzoate, (B) L-malate and benzoate, (C) oxaloacetate and benzoate, and (D) 4-hydroxybenzoate and succinate. **Figure S3.** Anaerobic growth and substrate utilization profiles of *Magnetospirillum* sp. strain pMbN1 with binary substrate mixtures of (A) acetate and benzoate, (B) acetate and 4-methylbenzoate, (C) pyruvate and benzoate, and (D) pyruvate and 4-methylbenzoate. **Figure S4.** Exemplary 1DE and 2DE (2D DIGE) gel images from cells grown with the ternary substrate mixture. **Table S1.** Fold change in the abundance of soluble proteins during anaerobic growth of *Magnetospirillum* sp. strain pMbN1 with selected substrate mixtures. **Table S2.** Abundance changes (ratios) of selected transcripts in cells of *Magnetospirillum* sp. strain pMbN1 anaerobically grown with benzoate, 4-methylbenzoate or succinate (reference) as single substrates, or ternary substrate mixture. **Table S3.** Selected proteins identified from membrane protein-enriched fractions of *Magnetospirillum* sp. strain pMbN1 grown anaerobically with benzoate, 4-methylbenzoate and succinate as single substrates, or ternary mixture. **Table S4.** Annotation of genes related to anaerobic degradation of 4-methylbenzoate, benzoate and succinate in *Magnetospirillum* sp. strain pMbN1. **Table S5.** Anaerobic growth of *Magnetospirillum* sp. strain pMbN1 with aliphatic and aromatic acids supplied as single substrates. **Table S6.** Primers used for reverse transcription real-time PCR. **Table S7.** Average cycle threshold (C_T_) values and efficiencies (E) obtained from reverse transcription real-time PCR reactions.
