# Supplementary material for: Benzoate mediates the simultaneous repression of anaerobic 4-methylbenzoate and succinate utilization in Magnetospirillum sp. strain pMbN1
Source: BMC Microbiol. 2014 Oct 27;14:269. doi: 10.1186/s12866-014-0269-4 (PMC4268860; doi:10.1186/s12866-014-0269-4)
Supplement: Additional file 1: Figure S1. — Anaerobic growth and substrate utilization profiles of Magnetospirillum sp. strain pMbN1 with binary substrate mixtures of (A) succinate and 4-hydroxybenzoate, (B) succinate and phenylacetate, (C) succinate and acetate, and (D) 4-methylbenzoate and phenylacetate. Figure S2. Anaerobic growth and substrate utilization profiles of Magnetospirillum sp. strain pMbN1 with binary substrate mixtures of (A) fumarate and benzoate, (B) L-malate and benzoate, (C) oxaloacetate and benzoate, and (D) 4-hydroxybenzoate and succinate. Figure S3. Anaerobic growth and substrate utilization profiles of Magnetospirillum sp. strain pMbN1 with binary substrate mixtures of (A) acetate and benzoate, (B) acetate and 4-methylbenzoate, (C) pyruvate and benzoate, and (D) pyruvate and 4-methylbenzoate. Figure S4. Exemplary 1DE and 2DE (2D DIGE) gel images from cells grown with the ternary substrate mixture. Table S1. Fold change in the abundance of soluble proteins during anaerobic growth of Magnetospirillum sp. strain pMbN1 with selected substrate mixtures. Table S2. Abundance changes (ratios) of selected transcripts in cells of Magnetospirillum sp. strain pMbN1 anaerobically grown with benzoate, 4-methylbenzoate or succinate (reference) as single substrates, or ternary substrate mixture. Table S3. Selected proteins identified from membrane protein-enriched fractions of Magnetospirillum sp. strain pMbN1 grown anaerobically with benzoate, 4-methylbenzoate and succinate as single substrates, or ternary mixture. Table S4. Annotation of genes related to anaerobic degradation of 4-methylbenzoate, benzoate and succinate in Magnetospirillum sp. strain pMbN1. Table S5. Anaerobic growth of Magnetospirillum sp. strain pMbN1 with aliphatic and aromatic acids supplied as single substrates. Table S6. Primers used for reverse transcription real-time PCR. Table S7. Average cycle threshold (CT) values and efficiencies (E) obtained from reverse transcription real-time PCR reactions. [file 12866_2014_269_MOESM1_ESM.pdf]

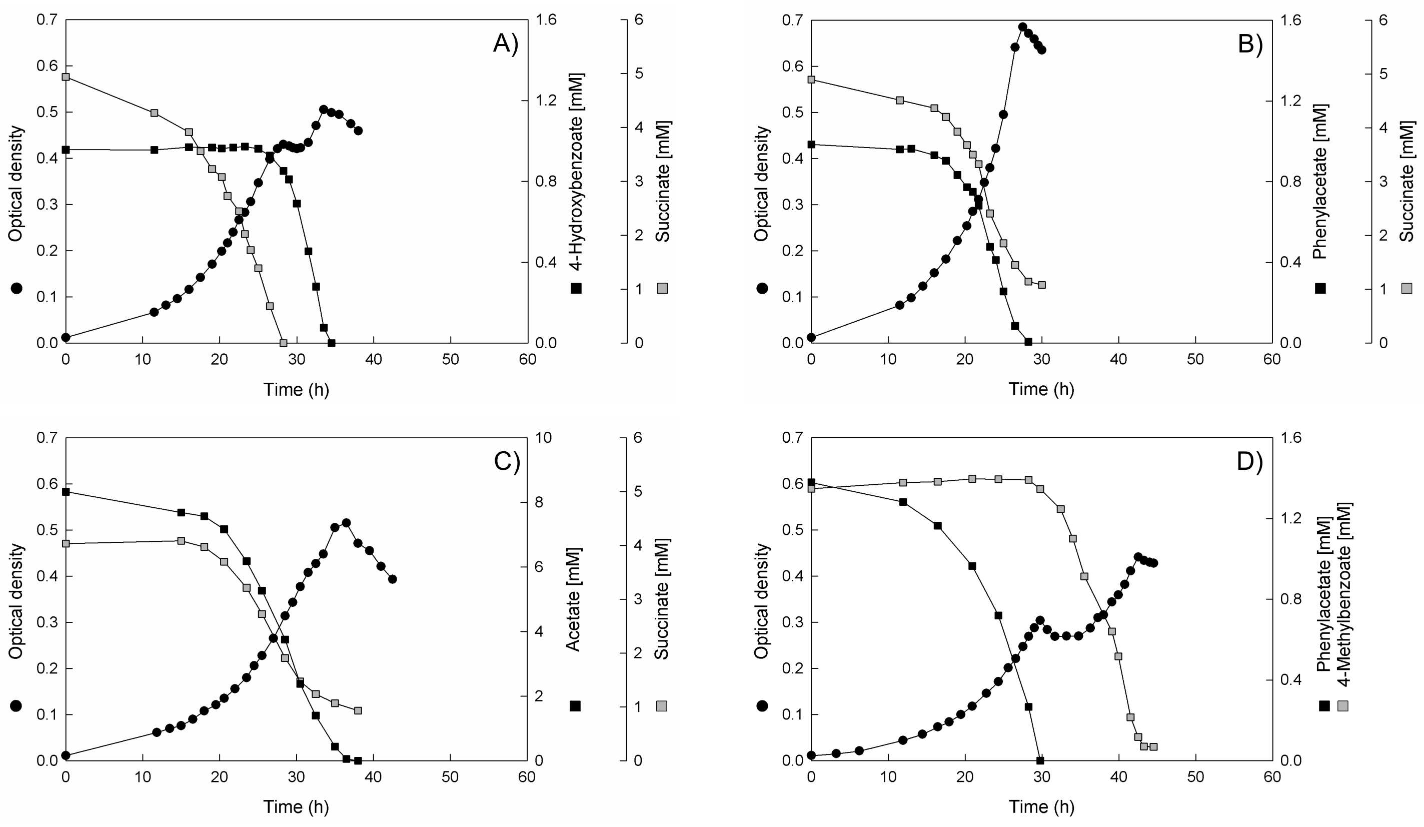

Figure S1 Anaerobic growth and substrate utilization profiles of *Magnetospirillum* sp. pMbN1 with binary substrate mixtures of (A) succinate and 4-hydroxybenzoate, (B) succinate and phenylacetate, (C) succinate and acetate, and (D) 4-methylbenzoate and phenylacetate. The respective adaptation substrate is always listed first.

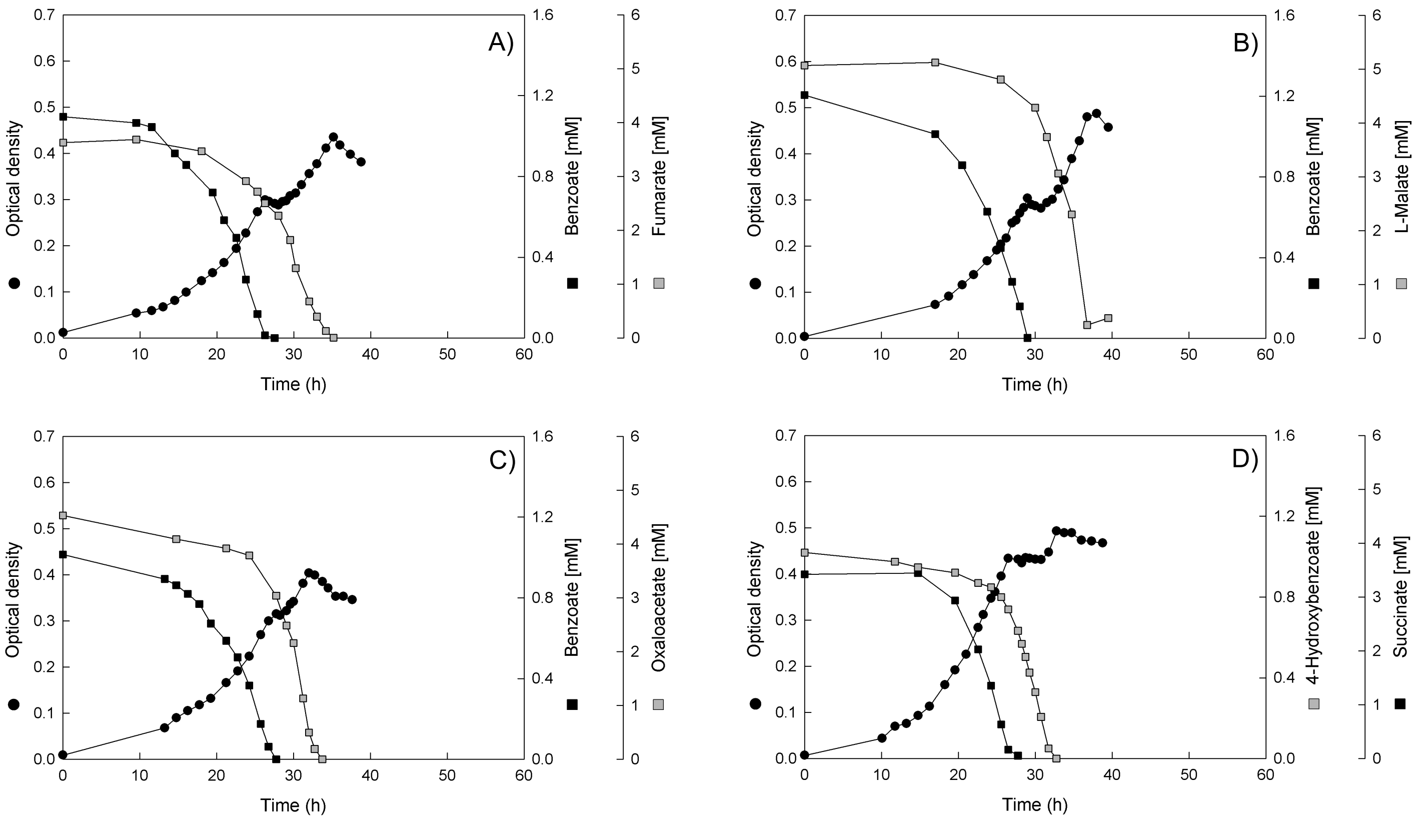

Figure S2 Anaerobic growth and substrate utilization profiles of *Magnetospirillum* sp. pMbN1 with binary substrate mixtures of (A) fumarate and benzoate, (B) L-malate and benzoate, (C) oxaloacetate and benzoate, and (D) 4-hydroxybenzoate and succinate. The respective adaptation substrate is always listed first.

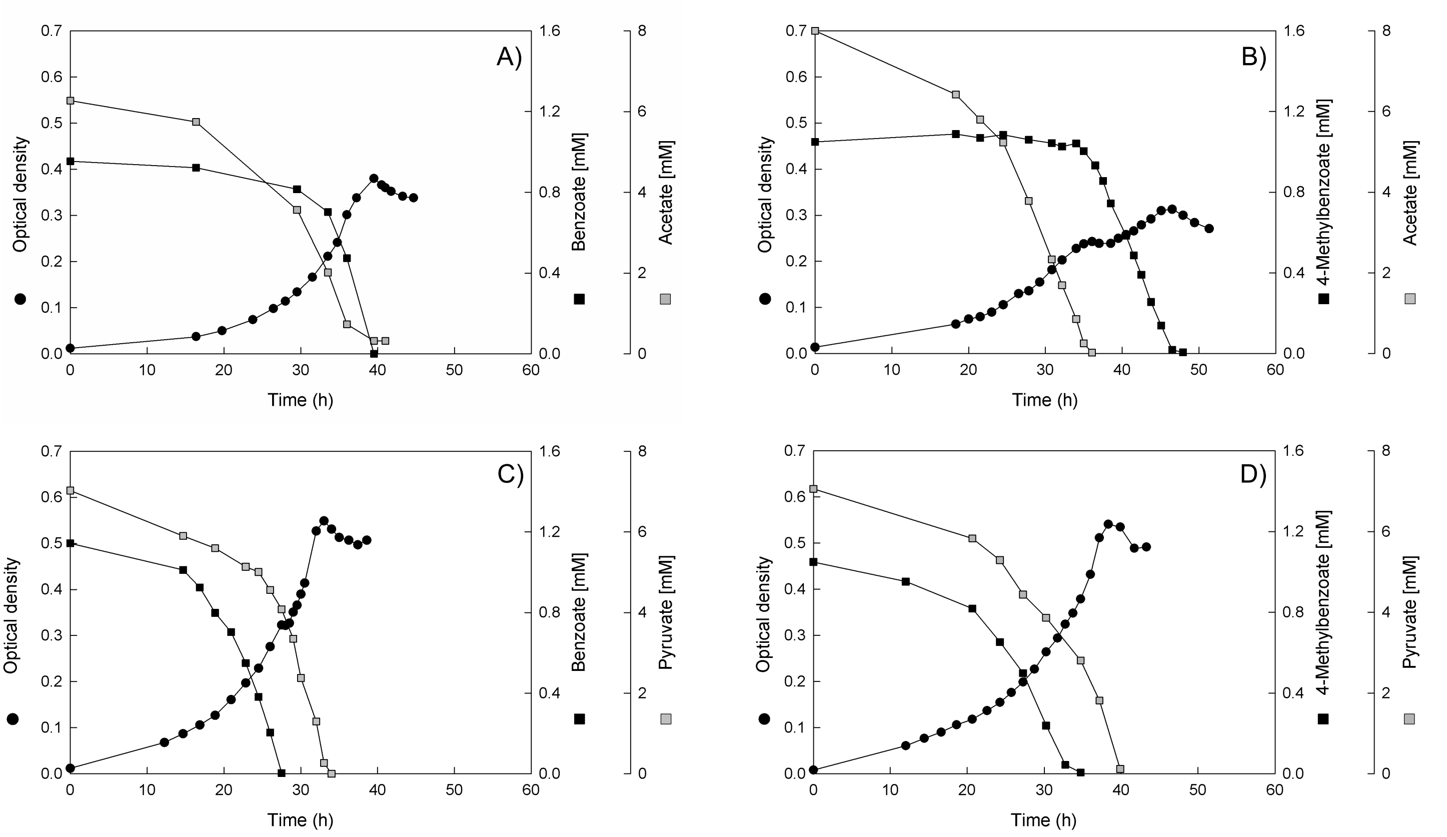

Figure S3 Anaerobic growth and substrate utilization profiles of *Magnetospirillum* sp. pMbN1 with binary substrate mixtures of (A) acetate and benzoate, (B) acetate and 4-methylbenzoate, (C) pyruvate and benzoate, and (D) pyruvate and 4-methylbenzoate. The respective adaptation substrate is always listed first.

Table S1 Fold change in the abundance of soluble proteins during anaerobic growth of *Magnetospirillum* sp. pMbN1 with selected substrate mixtures (reference: succinate-adapted cells).

		Fold change in abundance compared to that of cells grown with succinate[b]														
		Single substrate[c]		Substrate mixture[c]												
				S + B			S + M		M + B			M + B + S				
Protein[a]	Predicted function[a]	B	M	Ph1	Ph2	Ph3	Ph1a	Ph1b	Ph1	Ph2	Ph3	Ph1	Ph2a	Ph2b	Ph3a	Ph3b
Proteins related to anaerobic benzoate degradation																
BclA	Benzoate-CoA ligase	**5.2**	**3.7**	**3.4**	**3.2**	**2.8**	2.2	**2.8**	**5.1**	**4.9**	**3.7**	**4.7**	**4.6**	**4.2**	**3.7**	**3.3**
BcrA	Benzoyl-CoA reductase, α-subunit	**29.3**	**20.0**	**18.8**	**18.4**	**12.3**	**9.5**	**12.7**	**29.0**	**27.8**	**23.3**	**24.3**	**26.1**	**21.7**	**19.5**	**16.9**
BcrB	Benzoyl-CoA reductase, β-subunit	**18.7**	**9.4**	**12.1**	**11.0**	**8.1**	**4.5**	**7.0**	**17.1**	**15.9**	**12.0**	**15.4**	**15.2**	**13.1**	**11.0**	**9.6**
BcrC	Benzoyl-CoA reductase, γ-subunit	**5.4**	**3.4**	**3.9**	**3.7**	**2.9**	1.7	**2.6**	**5.6**	**5.2**	**4.1**	**5.0**	**4.8**	**4.4**	**3.7**	**3.3**
BcrD	Benzoyl-CoA reductase, δ-subunit	**16.7**	**8.0**	**11.5**	**10.3**	**7.6**	**3.7**	**5.6**	**15.7**	**14.1**	**11.0**	**14.0**	**14.0**	**11.5**	**9.9**	**8.2**
Dch-1	Cyclohex-1,5-diene-1-carbonyl-CoA hydratase	**17.6**	**9.8**	**12.2**	**10.6**	**8.1**	**4.8**	**7.1**	**16.9**	**15.2**	**12.3**	**15.1**	**15.0**	**13.4**	**11.5**	**10.2**
Had-1	6-Hydroxycyclohex-1-ene-1-carbonyl-CoA dehydrogenase	**3.3**	**2.9**	**3.1**	**2.9**	**2.5**	1.8	2.0	**4.2**	**3.9**	**3.3**	**3.6**	**3.5**	**3.3**	**2.8**	**2.8**
Oah-1	6-Oxocyclohex-1-ene-1-carbonyl-CoA hydrolase	**8.9**	**5.7**	**6.5**	**5.8**	**4.8**	**2.9**	**4.0**	**8.9**	**8.4**	**7.2**	**7.8**	**7.5**	**7.0**	**6.6**	**5.9**
KorA	2-Oxoacid:ferredoxin oxidoreductase; α-subunit	2.1	2.3	1.6	1.6	1.5	1.6	1.8	1.9	2.0	2.2	1.7	1.7	1.8	1.8	2.0
KorB	2-Oxoacid:ferredoxin oxidoreductase; β-subunit	2.0	1.8	1.5	1.6	1.5	1.4	1.6	1.8	1.8	2.0	1.6	1.6	1.7	1.7	1.7
Proteins related to uptake and anaerobic degradation of 4-methylbenzoate																
Orf870	ABC-type transport system, periplasmic solute-binding protein	−1.6	**6.2**	1.2	−1.2	1.7	**4.0**	**4.7**	1.5	**6.4**	**13.6**	2.1	1.4	**3.8**	**6.0**	**5.7**
MclA	Putative 4-methylbenzoate-CoA ligase	−1.1	**7.2**	−1.2	1.1	1.2	**4.6**	**5.1**	1.5	**3.2**	**4.1**	1.6	1.3	**2.9**	**4.0**	**4.9**
MbrA	4-Methylbenzoyl-CoA reductase, α-subunit	−1.3	**113.8**	−1.8	−1.0	−1.8	**47.5**	**69.3**	**10.1**	**29.3**	**77.3**	**11.6**	**9.3**	**27.9**	**42.6**	**60.1**
MbrB	4-Methylbenzoyl-CoA reductase, β-subunit	**4.2**	**54.0**	2.3	**2.8**	**2.9**	**25.3**	**30.7**	**7.3**	**14.9**	**30.4**	**10.4**	**6.7**	**16.0**	**22.6**	**30.9**
MbrC	4-Methylbenzoyl-CoA reductase, γ-subunit	1.2	**47.1**	1.5	1.3	1.4	**20.4**	**27.4**	**6.1**	**12.1**	**27.9**	**6.5**	**5.5**	**13.3**	**19.3**	**27.4**
MbrD	4-Methylbenzoyl-CoA reductase, δ-subunit	1.3	**12.0**	1.2	1.2	1.0	**9.4**	**17.7**	1.8	**3.7**	**9.2**	1.9	1.8	**4.3**	**6.4**	**7.5**
Dch-2	4-Methylcyclohex-1,5-diene-1-carbonyl-CoA	1.5	**4.1**	1.2	1.4	1.4	**3.1**	**3.2**	1.6	1.8	**2.5**	1.7	1.6	1.9	2.2	**2.6**
Had-2	4-Methyl-6-hydroxycyclohex-1-ene-1-carbonyl-CoA dehydrogenase	−1.1	1.8	−1.0	1.0	1.1	1.4	1.5	1.0	1.2	1.3	1.1	1.0	1.2	1.3	1.3
Oah-2	4-Methyl-6-oxocyclohex-1-ene-carbonyl-CoA hydrolase	1.6	**86.3**	1.2	−1.3	−1.1	**48.1**	**61.5**	**10.6**	**22.5**	**39.6**	**10.4**	**9.0**	**22.6**	**31.5**	**42.5**
Proteins related to succinate uptake and catabolism																
DctP	Periplasmic solute-binding protein of C_4-dicarboxylate TRAP transporter	**−5.0**	**−6.0**	−2.1	−1.5	1.4	−1.7	−1.9	**−5.7**	**−4.8**	**−4.4**	**−3.4**	**−3.4**	−2.4	−2.0	−1.7
PdhB	Pyruvate dehydrogenase, E1 component	**−11.8**	**−10.5**	**−3.7**	−2.1	−1.1	−1.5	−1.9	**−9.9**	**−9.1**	**−7.5**	**−9.9**	**−10.3**	**−2.7**	−2.4	**−2.6**
LpdA	Pyruvate dehydrogenase, E3 component	**−6.6**	**−6.2**	**−2.9**	−2.0	−1.2	−1.4	−1.9	**−4.9**	**−5.6**	**−5.3**	**−6.2**	**−7.0**	**−2.5**	−2.4	**−2.5**

[a] For accession numbers and functional annotation of respective proteins see Additional file 1: Table S4.

[b] Fold changes in protein abundance as determined by 2D DIGE. Values marked in bold were above the applied threshold for significantly changed abundances (>|2.5|).

[c] Abbreviations: B, benzoate; M, 4-methylbenzoate; S, succinate; Ph, phase. For further details see Figure 3.

Table S2 Abundance changes (ratios) of selected transcripts in cells of *Magnetospirillum* sp. pMbN1 anaerobically grown with benzoate, 4-methyl-benzoate or succinate (reference) as single substrates, or ternary substrate mixture (see Figures 1D and 2).

	Ratio in transcript abundance[a]											
	Single substrates		Mixture of 4-methylbenzoate, benzoate and succinate (sampling points with cultivation time after inoculation)									
			Benzoate utilization phase (phase 1)					Diauxic lag phase (phase 2)		4-Methylbenzoate and bulk succinate utilization phase (phase 3)		
Gene name	Benzoate	4-Methyl-benzoate	14.8 h	19.9 h	20.9 h	21.9 h	22.9 h	23.4 h	24.9 h	26.4 h	28.0 h	28.9 h
Genes related to benzoate utilization												
bclA	8 ± 3	3 ± 2	6 ± 3	4 ± 1	5 ± 0	6 ± 2	5 ± 1	3 ± 1	1 ± 1	1 ± 0	2 ± 0	4 ± 1
bcrC	69 ± 25	34 ± 14	53 ± 21	48 ± 22	61 ± 16	81 ± 26	60 ± 7	13 ± 3	2 ± 0	5 ± 1	8 ± 1	16 ± 5
benK	6 ± 3	2 ± 1	2 ± 1	2 ± 1	3 ± 0	3 ± 1	2 ± 0	1 ± 1	−1 ± 0	−1 ± 0	−1 ± 0	2 ± 0
Genes related to 4-methylbenzoate utilization												
mclA	−2 ± 1	282 ± 78	3 ± 1	3 ± 1	5 ± 1	9 ± 1	16 ± 5	139 ± 45	282 ± 51	209 ± 26	230 ± 22	394 ± 139
mbrC	−1 ± 1	396 ± 109	2 ± 0	2 ± 1	3 ± 1	6 ± 2	7 ± 1	126 ± 45	596 ± 86	548 ± 93	571 ± 87	1046 ± 235
orf870	−2 ± 1	53 ± 16	2 ± 1	2 ± 0	2 ± 0	2 ± 0	6 ± 1	34 ± 15	108 ± 39	49 ± 11	57 ± 24	62 ± 29
Genes related to succinate utilization												
pdhB	−13 ± 6	−17 ± 8	−19 ± 8	−14 ± 3	−11 ± 3	−14 ± 1	−16 ± 4	−19 ± 6	−1 ± 1	−5 ± 1	−12 ± 3	−7 ± 2
maeB	−9 ± 5	−11 ± 6	−5 ± 2	−5 ± 1	−4 ± 1	−4 ± 1	−3 ± 1	−3 ± 1	−2 ± 0	−2 ± 1	−3 ± 1	−1 ± 1
dctP	−18 ± 8	−19 ± 7	−6 ± 2	−8 ± 3	−4 ± 0	−4 ± 2	−4 ± 2	−4 ± 1	−2 ± 0	−2 ± 0	−2 ± 0	−1 ± 2
dctM	−14 ± 4	−15 ± 6	−5 ± 2	−6 ± 0	−5 ± 1	−4 ± 1	−3 ± 1	−3 ± 1	−1 ± 0	−2 ± 0	−2 ± 1	−1 ± 1
dctA	−18 ± 7	−26 ± 12	−6 ± 3	−6 ± 2	−3 ± 1	−4 ± 1	−3 ± 1	−5 ± 2	−4 ± 2	−3 ± 1	−3 ± 1	−2 ± 1

[a] Ratio of transcript abundance between test and reference (succinate) state. Data used for calculation of transcript abundances is provided in Table S7.

Ratio: > 500 | > 250 | > 50 | > 10 | ≥ 5 | > −5 | ≤ −5 | < −10 | < −20

Table S3 Selected proteins identified from membrane protein-enriched fractions of *Magnetospirillum* sp. pMbN1 grown anaerobically with benzoate, 4-methylbenzoate and succinate as single substrates, or ternary mixture (for 1DE gel image see Additional file 1: Figure S4).

Protein name[a]	Predicted function[a]	Single substrates			Ternary substrate mixture		
		Benzoate	4-Methyl-benzoate	Succinate	Phase 1	Phase 2b	Phase 3b
Proteins related to benzoate uptake							
BenK	Benzoate/H^+ symporter	476	277		304	325	301
Proteins related to 4-methylbenzoate uptake							
Orf960	ABC-type transport system, permease component						100
Orf950	ABC-type transport system, permease component		74			62	185
Orf930	ABC-type transport system, ATPase component		115				58
Orf920	ABC-type transport system, ATPase component		153			46	133
Proteins related to C_4-dicarboxylate uptake and degradation							
DctQ	TRAP-type C_4-dicarboxylate transport system, permease	82		252		129	157
DctM	TRAP-type C_4-dicarboxylate transport system, permease			98	63		
DctA	C_4-dicarboxylate/Na^+ symporter			254	65	113	137
MaeB	$NADP^+$-dependent malic enzyme			93	89		
PdhA	Pyruvate dehydrogenase, E1 component, α-subunit			45			
Central metabolism							
GltA	Citrate synthase	114	93		226	99	
AcnB	Aconitase	80	127		126		
Icd	Isocitrate dehydrogenase	244	284		545	174	147
SucC	Succinyl-CoA ligase, β-subunit	269	297	217	500	252	281
SucD	Succinyl-CoA ligase, α-subunit		76		151	75	
SdhB	Succinate dehydrogenase, β-subunit				35		
SdhC	Succinate dehydrogenase, γ-subunit	206	166	122		136	172
SdhD	Succinate dehydrogenase, δ-subunit	102	102	93	35	73	118
FumA	Fumarate hydratase class I	126			249		
Mdh	Malate dehydrogenase	305	241	173	398	290	178

Mascot score:	not detected	> 20	> 200	> 500

[a] For accession numbers and functional annotation of respective proteins see Additional file 1: Table S4.

Table S4 Anntotation of genes related to anaerobic degradation of 4-methylbenzoate, benzoate and succinate in *Magnetospirillum* sp. pMbN1 .

Gene identifier	Status[a]	Accession No.[b]	Protein name	Length (amino acids)	INTERPRO	relevant BLASTP hit[c]					Predicted function
						Gene name	Organism	Identity	e-Value	Accession no.[d]	
**Genes related to anaerobic 4-methylbenzoate utilization**											
pmbn1w00890	F	KF941519	MclA	549	IPR000873	*bclA*	*Thauera aromatica*	26%	6×10^{-24}	Q8GQN9	4-Methylbenzoate-CoA ligase
pmbn1w00500	F	KF941520	MbrC	390	IPR010327	*bcrC*	*Thauera aromatica*	33%	1×10^{-59}	O87874	4-Methylbenzoyl-CoA reductase, γ–subunit
pmbn1w00490	F	KF941521	MbrB	450	IPR010327	*bcrB*	*Thauera aromatica*	33%	3×10^{-46}	O87875	4-Methylbenzoyl-CoA reductase, β–subunit
pmbn1w00510	F	KF941522	MbrA	269	IPR002731, IPR008275	*bcrA*	*Thauera aromatica*	46%	3×10^{-49}	O87876	4-Methylbenzoyl-CoA reductase, α–subunit
pmbn1w00520	F	KF941523	MbrD	291	IPR002731, IPR008275	*bcrD*	*Thauera aromatica*	33%	1×10^{-32}	O87877	4-Methylbenzoyl-CoA reductase, δ–subunit
pmbn1w00830	F	KF941524	Dch-2	257	IPR001753, IPR017602	*dch*	*Thauera aromatica*	64%	9×10^{-80}	O87873	4-Methylcyclohex-1,5-diene-1-carbonyl-CoA hydratase
pmbn1w00850	F	KF941525	Had-2	324	IPR002085, IPR002328, IPR011032, IPR013149, IPR013154, IPR016040, IPR017614	*had*	*Thauera aromatica*	64%	1×10^{-103}	O87871	6-Hydroxy-4-methylcyclohex-1-ene-1-carbonyl-CoA dehydrogenase
pmbn1w00840	F	KF941526	Oah-2	379	IPR001753, IPR017613,	*oah*	*Thauera aromatica*	75%	1×10^{-172}	O87872	4-Methyl-6-oxocyclohex-1-ene-1-carbonyl-CoA hydrolase
pmbn1w00880	F	KF941527	Orf880	222	IPR001647, IPR009057, IPR011075, IPR015893, IPR023772	*mb3439c*	*Mycobacterium bovis*	37%	3×10^{-7}	P67443	TetR-type transcriptional regulator
pmbn1w00870	F	KF941528	Orf870	381	IPR028081, IPR028082	*amb1552*	*Magnetospirillum magneticum*	60%	7×10^{-156}	Q2W719	ABC-type transport system, periplasmic solute-binding protein
pmbn1w00920	F	KF941529	Orf920	266	IPR003439, IPR003593, IPR017871, IPR027417	*livF*	*Salmonella typhimurium*	51%	8×10^{-70}	P0A191	ABC-type transport system, ATP-binding protein
pmbn1w00930	F	KF941530	Orf930	271	IPR003439, IPR003593, IPR017871, IPR027417	*braF*	*Pseudomonas aeruginosa*	42%	4×10^{-65}	P21629	ABC-type transport system, ATP-binding protein
pmbn1w00950	F	KF941531	Orf950	290	IPR001851	*livH*	*Salmonella typhimurium*	31%	6×10^{-47}	P0A2J1	ABC-type transport system, permease component
pmbn1w00960	F	KF941532	Orf960	348	IPR001851	*braE*	*Pseudomonas aeruginosa*	33%	4×10^{-47}	P21628	ABC-type transport system, permease component
**Genes related to anaerobic benzoate utilization**											
pmbn1C394_0012	F	KF941495	BclA	528	IPR000873, IPR025110, IPR011957	*bclA*	*Thauera aromatica*	60%	0.0	Q8GQN9	Benzoate-CoA ligase (EC 6.2.1.25)
pmbn1w00160	F	KF941533	BcrC	391	IPR010327, IPR011955	*bcrC*	*Thauera aromatica*	62%	1×10^{-154}	O87874	Benzoyl-CoA reductase, γ–subunit
pmbn1w00150	F	KF941534	BcrB	432	IPR010327, IPR011955	*bcrB*	*Thauera aromatica*	75%	0.0	O87875	Benzoyl-CoA reductase, β–subunit
pmbn1w00140	F	KF941535	BcrA	430	IPR002731, IPR008275, IPR011954	*bcrA*	*Thauera aromatica*	72%	1×10^{-178}	O87876	Benzoyl-CoA reductase, α–subunit
pmbn1w00130	F	KF941536	BcrD	279	IPR002731, IPR008275, IPR011956	*bcrD*	*Thauera aromatica*	74%	1×10^{-107}	O87877	Benzoyl-CoA reductase, δ–subunit
pmbn1w00120	F	KF941537	Fdx	79	IPR017896, IPR017900	*fdx*	*Thauera aromatica*	60%	6×10^{-15}	O88151	Ferredoxin
pmbn1w00200	F	KF941538	KorA	578	IPR002869, IPR002880, IPR005476, IPR009014, IPR015941, IPR019752, IPR022367	*korA*	*Thauera aromatica*	60%	0.0	O87870	2-Oxoglutarate:ferredoxin oxidoreductase, α–subunit
pmbn1w00210	F	KF941539	KorB	279	IPR011766	*korB*	*Thauera aromatica*	67%	1×10^{-111}	Q8RJQ9	2-Oxoglutarate:ferredoxin oxidoreductase, β–subunit
pmbn1w00170	F	KF941540	Dch-1	259	IPR001753, IPR017602	*dch*	*Thauera aromatica*	66%	4×10^{-82}	O87873	Cyclohex-1,5-diene-1-carbonyl-CoA hydratase
pmbn1w00190	F	KF941541	Had-1	358	IPR002085, IPR002328, IPR011032, IPR013149, IPR013154, IPR016040, IPR017614	*had*	*Thauera aromatica*	63%	1×10^{-114}	O87871	6-Hydroxycylohex-1-ene-1-carbonyl-CoA dehydrogenase
pmbn1w00180	F	KF941542	Oah-1	377	IPR001753, IPR017613	*oah*	*Thauera aromatica*	79%	1×10^{-179}	O87872	6-Oxocyclohex-1-ene-1-carbonyl-CoA hydratase
pmbn1w04893	D	KF941494	BenK	453	IPR005829, IPR011701, IPR016196, IPR020846	*benK*	*Acinetobacter sp.*	49%	1×10^{-152}	O30513	Benzoate/ H^+ symporter
**Genes related to succinate utilization**											
pmbn1C3_00100	F	KF941496	DctP	337	IPR004682, IPR018389	*dctP*	*Rhodobacter capsulatus*	69%	6×10^{-153}	P37735	TRAP-type periplasmic C_4-dicarboxylate-binding protein
pmbn1C3_00110	F	KF941497	DctQ	233	IPR007387	*dctQ*	*Rhodobacter capsulatus*	52%	7×10^{-64}	D5AQF8	TRAP-type C_4-dicarboxylate transport system, permease component
pmbn1C3_00120	F	KF941498	DctM	427	IPR004681, IPR010656	*dctM*	*Rhodobacter capsulatus*	77%	0.0	D5AQF7	TRAP-type C_4-dicarboxylate transport system, permease component

Table S4 continued

						relevant BLASTP hit[c]					
Gene identifier	Status[a]	Accession No.[b]	Protein name	Length (amino acids)	INTERPRO	Gene name	Organism	Identity	e-Value	Accession no.[d]	Predicted function
pmbn1C3_00090	F	KF941499	DctS	651	IPR000014, IPR000700, IPR001610, IPR003594, IPR003661, IPR005467, IPR009082, IPR013767, IPR004358	*dctS*	*Rhodobacter capsulatus*	42%	2×10^{-149}	P37739	Sensory histidine kinase of C_4-dicarboxylate-responsive sensory/regulatory system
pmbn1C3_00080	F	KF941500	DctR	200	IPR000792, IPR001789, IPR011006, IPR011991	*dctR*	*Rhodobacter capsulatus*	51%	6×10^{-58}	P37740	DNA-binding response regulator of C_4-dicarboxylate-responsive sensory/regulatory system
pmbn1w02526	D	KF941501	DctA	415	IPR001991, IPR018107	*dctA*	*Sinorhizobium meliloti*	63%	4×10^{-172}	P20672	C_4-dicarboxylate/ Na^+ symporter
pmbn1w03530	D	KF941502	DctB	581	IPR003594, IPR003661, IPR005467, IPR009082, IPR017055, IPR004358	*dctB*	*Rhizobium leguminosarum*	34%	1×10^{-93}	P10047	Sensory histidine kinase of C_4-dicarboxylate-responsive sensory/regulatory system
pmbn1w3533	D	KF941503	DctD	446	IPR001789, IPR002078, IPR002197, IPR003593, IPR009057, IPR011006, IPR025943, IPR025944, IPR027417	*dctD*	*Sinorhizobium meliloti*	48%	3×10^{-137}	P13632	DNA-binding response regulator of C_4-dicarboxylate-responsive sensory/regulatory system
pmbn1d03865	D	KF941505	PdhA	332	IPR017597, IPR001017	*pdhA*	*Sinorhizobium meliloti*	68%	3×10^{-154}	Q9R9N5	Pyruvate dehydrogenase, E1 component, α–subunit
pmbn1d03867	D	KF941506	PdhB	440	IPR000089, IPR003016, IPR005475, IPR005476, IPR009014, IPR011053, IPR015941, IPR027110	*pdhB*	*Sinorhizobium meliloti*	63%	0.0	Q9R9N4	Pyruvate dehydrogenase, E1 component, β–subunit
pmbn1d03876	D	KF941507	LpdA	462	IPR004099, IPR006258, IPR012999, IPR013027, IPR016156, IPR023753	*lpdA*	*Rhizobium etli*	61%	0.0	O05940	Pyruvate dehydrogenase, E3 component
pmbn1w00775	D	KF941504	MaeB	762	IPR002505, IPR012188, IPR012301, IPR012302, IPR016040	*maeB*	*Escherichia coli*	62%	0.0	P76558	$NADP^+$-dependent malic enzyme
**Genes related to central metabolism**											
pmbn1w00530	D	KF941508	GltA	438	IPR002020, IPR010953, IPR016141, IPR016142, IPR016143, IPR019810, IPR024176	*gltA*	*Sinorhizobium meliloti*	73%	0.0	O33915	Citrate synthase (EC 2.3.3.1)
pmbn1w03702	D	KF941509	AcnB	861	IPR001030, IPR004406, IPR015928, IPR015929, IPR015930, IPR015931, IPR015932 IPR018136 IPR015933, IPR015937	*acnB*	*Pseudomonas aeruginosa*	73%	0.0	Q9I2V5	Aconitase 2 (EC 4.2.1.3)
pmbn1w00603	D	KF941510	Icd	741	IPR004436, IPR024084	*icd*	*Azotobacter vinelandii*	75%	0.0	P16100	Isocitrate dehydrogenase (EC 1.1.1.42)
pmbn1w06208	D	KF941511	SucC	398	IPR005809, IPR005811, IPR011761, IPR013650, IPR013815, IPR013816, IPR016102	*sucC*	*Magnetospirillum magneticum*	97%	0.0	Q2W063	Succinyl-CoA ligase, β–subunit (EC 6.2.1.5)
pmbn1w06210	D	KF941512	SucD	291	IPR003781, IPR005810, IPR005811, IPR016040, IPR016102, IPR017440	*sucD*	*Rickettsia bellii*	78%	5×10^{-166}	Q1RH56	Succinyl-CoA ligase, α–subunit (EC 6.2.1.5)
pmbn1w04352	D	KF941513	SdhA	593	IPR003952, IPR003953, IPR011281, IPR014006, IPR015939, IPR027477	*sdhA*	*Paracoccus denitrificans*	72%	0.0	Q59661	Succinate dehydrogenase, flavoprotein subunit (EC 1.3.99.1)
pmbn1w04353	D	KF941514	SdhB	259	IPR001041, IPR004489, IPR006058, IPR009051, IPR012285, IPR012675, IPR017900, IPR017896, IPR025192	*sdhB*	*Rickettsia bellii*	72%	4×10^{-139}	Q1RGP3	Succinate dehydrogenase, iron-sulfur subunit
pmbn1w04350	D	KF941515	SdhC	109	IPR000701, IPR014314, IPR014361, IPR018495	*sdhC*	*Paracoccus denitrificans*	45%	4×10^{-24}	Q59659	Succinate dehydrogenase, cytochrome b_{556} subunit
pmbn1w04351	D	KF941516	SdhD	126	IPR000701, IPR014312	*sdhD*	*Rickettsia bellii*	39%	8×10^{-19}	Q1RHB6	Succinate dehydrogenase, hydrophobic membrane anchor subunit
pmbn1w01837	D	KF941517	FumA	538	IPR004646, IPR004647, IPR011167, IPR020557	*fumA*	*Salmonella typhimurium*	67%	0.0	P40720	Fumarate hydratase class I (Fumarase) (EC 4.2.1.2)
pmbn1w06206	D	KF941518	Mdh	319	IPR001236, IPR001557, IPR011275, IPR015955, IPR016040, IPR022383	*mdh*	*Magnetospirillum magneticum*	99%	0.0	Q2W064	Malate dehydrogenase (EC 1.1.1.37)

[a] Status of DNA sequence: F, finished; D, draft.

[b] GenBank accession number.

[c] Proteins related to the anaerobic degradation of 4-methylbenzoate and benzoate were compared to protein sequences of biochemically characterized proteins of *Thauera aromatica* K172.

[d] UniProtKB/Swiss-Prot accession number.

Table S5 Anaerobic growth of *Magnetospirillum* sp. pMbN1 with aliphatic and aromatic acids supplied as single substrates.

From experiment with binary mixture of:		Growth with single substrates			
		Adaptation substrate		Co-substrate	
**Adaptation substrate**	**Co-substrate**	**OD_{max}**	**μ_{max} (h^{-1})**	**OD_{max}**	**μ_{max} (h^{-1})**
Cells adapted to succinate					
Succinate (5 mM)	**Benzoate (1 mM)**	0.38	0.21	0.18	0.21
Succinate (5 mM)	4-Methylbenzoate (1 mM)	0.38	0.21	0.15	0.13
**Succinate (5 mM)**	4-Hydroxybenzoate (1 mM)	0.36	0.20	ND	ND
Succinate (5 mM)	Phenylacetate (1 mM)	0.36	0.20	0.19	0.16
Succinate (5 mM)	Acetate (8 mM)	0.36	0.20	0.30	0.22
Cells adapted to 4-methylbenzoate					
4-Methylbenzoate (1.5 mM)	**Benzoate (1.5 mM)**	0.27	0.16	0.28	0.15
4-Methylbenzoate (1.5 mM)	**Phenylacetate (1.5 mM)**	0.27	0.16	0.32	0.16
Cells adapted to other aliphatic acids					
Acetate (8 mM)	Benzoate (1 mM)	0.27	0.18	0.17	0.20
**Acetate (8 mM)**	4-Methylbenzoate (1 mM)	0.27	0.18	0.18	0.19
Pyruvate (6 mM)	**Benzoate (1 mM)**	0.34	0.18	0.18	0.21
Pyruvate (6 mM)	4-Methylbenzoate (1 mM)	0.34	0.18	0.19	0.16
Fumarate (5 mM)	**Benzoate (1 mM)**	0.37	0.27	0.19	0.17
L-Malate (5 mM)	**Benzoate (1 mM)**	0.37	0.33	0.16	0.25
Oxaloacetate (6 mM)	**Benzoate (1 mM)**	0.27	0.20	0.17	0.23
Cells adapted to other aromatic acids					
4-Hydroxybenzoate (1 mM)	**Succinate (5 mM)**	0.13	0.20	0.34	0.10

Preferentially utilized substrates are highlighted in boldface (see Table 1). Applied substrate concentrations are indicated in parentheses. Values for maximal optical density (OD_{max}) and maximum specific growth rates (μ_{max}) are based on at least two replicate cultures yielding a standard deviation of <5%. μ_{max} was calculated from the slope of the active growth phase (*m*): $\mu_{max} = m \times 1/\Delta OD$. ND, not determined.

Table S6 Primers used for reverse transcription real-time PCR.

Primer	Sequence (5′→ 3′)	Target gene	Product length (bp)	Real-time detection temperature (°C)
benK 391F<br>benK 550R	GTGGCGCTGATGACCGAATACG<br>GAACCGCCGCCACGAAGAACAC	*benK*	160	84
bcrC 809F<br>bcrC 963R	CAGCTTCCAGATGCCCGACAGGTT<br>TCATCCACGGCGACGCTTACTATCA	*bcrC*	155	83
bclA 125F<br>bclA 276R	CCGACGGCAGCCACACCTAC<br>CTTGACGGCGCCCCAGAAC	*bclA*	152	83
orf870 457F<br>orf870 615R	TCGGGGCGTGAGCAGAC<br>GGCCCCCGTCCCGAATAC	*orf870*	159	82
mbrC 723F<br>mbrC 872R	ATAAAATGCGGGTGGTGTTGGAAGG<br>GGTCGCCCGCAGTATTGATGTCC	*mbrC*	150	83
mclA 217F<br>mclA 399R	CGCTTCGCCCGTGGTCTCTAC<br>GCCCGCATGTCGCAATAAGGTC	*mclA*	183	83
dctA 728F<br>dctA 879R	TGCTGGTCGGCTTCAACATCTTCA<br>CACCAGGCCGACCACCGACTT	*dctA*	152	84
dctP 319F<br>dctP 495R	TCTTCGACCTGCCCTACATCTTCC<br>GTCGGCGGGGGTCTTGAGC	*dctP*	176	84
dctM 181F<br>dctM 344R	TTCTTCATCCTGGCGGGCAACTT<br>ACGGTGGCGGGGCTGGAG	*dctM*	164	87
pdhB 716F<br>pdhB 877R	GCGCCCAGCACAGCCAGGATTA<br>TCTGGCCGTAGAGCAGTTCGTTTTC	*pdhB*	161	84
maeB 1121F<br>maeB 1286R	CCTTCGGGCCGGAATACCTGA<br>CCGAGCGGAACACGAACTGGT	*maeB*	165	84

Table S7 Average cycle threshold (C_T) values and efficiencies (E) obtained from reverse transcription real-time PCR reactions.

Gene name	Average C_T values and efficiencies[a]:																									
	Single substrate						Ternary mixture of 4-methylbenzoate, benzoate and succinate (cultivation time after inoculation)																			
							Benzoate utilization phase (phase 1)										Diauxic lag phase (phase 2)				4-Methylbenzoate and bulk succinate utilization phase (phase 3)					
	Benzoate		4-Methyl-benzoate		Succinate		14.8 h		19.9 h		20.9 h		21.9 h		22.9 h		23.4 h		24.9 h		26.4 h		28.0 h		28.9 h	
	C_T	E	C_T	E	C_T	E	C_T	E	C_T	E	C_T	E	C_T	E	C_T	E	C_T	E	C_T	E	C_T	E	C_T	E	C_T	E
bclA	24.4<br>±1.0	1.79<br>±0.04	25.5<br>±0.9	1.81<br>±0.04	27.4<br>±0.6	1.78<br>±0.06	24.4<br>±0.7	1.72<br>±0.05	25.1<br>±0.3	1.77<br>±0.06	24.8<br>±0.1	1.84<br>±0.07	24.5<br>±0.5	1.77<br>±0.05	24.6<br>±0.5	1.82<br>±0.06	25.4<br>±0.4	1.86<br>±0.05	27.5<br>±0.3	1.81<br>±0.03	27.1<br>±0.2	1.79<br>±0.04	26.4<br>±0.3	1.83<br>±0.06	25.1<br>±0.4	1.82<br>±0.07
bcrC	20.8<br>±0.8	1.85<br>±0.03	21.7<br>±0.6	1.83<br>±0.04	27.7<br>±0.6	1.77<br>±0.06	21.0<br>±0.6	1.74<br>±0.08	21.1<br>±0.6	1.78<br>±0.05	20.8<br>±0.4	1.86<br>±0.02	20.3<br>±0.5	1.77<br>±0.04	20.8<br>±0.2	1.73<br>±0.06	23.4<br>±0.4	1.88<br>±0.06	26.8<br>±0.0	1.83<br>±0.03	25.0<br>±0.3	1.79<br>±0.07	24.2<br>±0.3	1.77<br>±0.05	23.0<br>±0.5	1.88<br>±0.04
benK	24.4<br>±0.7	1.87<br>±0.05	26.1<br>±0.5	1.87<br>±0.07	27.2<br>±0.5	1.84<br>±0.07	25.8<br>±0.6	1.99<br>±0.04	26.1<br>±0.4	1.86<br>±0.07	25.7<br>±0.2	1.84<br>±0.03	25.5<br>±0.6	1.91<br>±0.06	26.0<br>±0.2	1.82<br>±0.05	26.9<br>±0.4	1.89<br>±0.08	27.8<br>±0.3	1.83<br>±0.05	27.7<br>±0.2	1.87<br>±0.05	27.9<br>±0.4	1.91<br>±0.06	26.6<br>±0.3	1.90<br>±0.04
mclA	29.9<br>±0.7	1.83<br>±0.05	19.9<br>±0.6	1.86<br>±0.04	28.7<br>±0.5	1.84<br>±0.06	26.9<br>±0.5	1.86<br>±0.03	26.8<br>±0.3	1.83<br>±0.04	26.2<br>±0.2	1.83<br>±0.04	25.2<br>±0.2	1.81<br>±0.02	24.3<br>±0.5	1.82<br>±0.06	20.7<br>±0.5	1.87<br>±0.01	19.6<br>±0.3	1.89<br>±0.07	20.1<br>±0.2	1.90<br>±0.02	20.0<br>±0.2	1.91<br>±0.05	19.0<br>±0.6	1.91<br>±0.03
mbrC	27.7<br>±1.0	1.81<br>±0.04	17.5<br>±0.4	1.98<br>±0.06	27.1<br>±0.7	1.80<br>±0.05	26.0<br>±0.2	1.83<br>±0.06	26.3<br>±0.9	1.84<br>±0.03	25.4<br>±0.3	1.87<br>±0.05	24.4<br>±0.5	1.80<br>±0.05	24.2<br>±0.1	1.78<br>±0.06	19.3<br>±0.5	1.80<br>±0.06	16.8<br>±0.2	1.94<br>±0.04	17.0<br>±0.3	1.90<br>±0.03	16.9<br>±0.2	1.88<br>±0.05	15.9<br>±0.4	1.90<br>±0.04
orf870	27.8<br>±0.7	1.85<br>±0.07	20.5<br>±0.7	1.90<br>±0.05	26.8<br>±0.7	1.74<br>±0.06	26.0<br>±0.6	1.80<br>±0.05	26.1<br>±0.3	1.86<br>±0.06	25.9<br>±0.1	1.82<br>±0.04	25.5<br>±0.1	1.83<br>±0.06	24.0<br>±0.2	1.88<br>±0.05	20.9<br>±0.7	1.79<br>±0.06	19.1<br>±0.6	1.81<br>±0.07	20.4<br>±0.4	1.86<br>±0.05	20.1<br>±0.6	1.87<br>±0.04	19.9<br>±0.8	1.75<br>±0.04
pdhB	25.6<br>±0.7	1.87<br>±0.06	26.1<br>±0.7	1.89<br>±0.06	21.5<br>±0.7	1.94<br>±0.07	26.2<br>±0.7	1.86<br>±0.04	25.8<br>±0.3	1.85<br>±0.06	25.4<br>±0.5	1.91<br>±0.04	25.8<br>±0.2	1.93<br>±0.06	26.0<br>±0.4	1.86<br>±0.06	26.3<br>±0.5	1.89<br>±0.04	22.2<br>±0.5	1.88<br>±0.02	24.1<br>±0.4	1.85<br>±0.06	25.6<br>±0.4	1.83<br>±0.07	24.8<br>±0.5	1.94<br>±0.04
maeB	25.9<br>±0.8	1.84<br>±0.04	26.1<br>±1.0	1.85<br>±0.04	22.3<br>±0.8	1.84<br>±0.05	24.8<br>±0.8	1.83<br>±0.04	25.1<br>±0.4	1.85<br>±0.07	24.7<br>±0.3	1.86<br>±0.05	24.5<br>±0.6	1.79<br>±0.03	24.3<br>±0.3	1.83<br>±0.06	24.0<br>±0.8	1.96<br>±0.05	23.2<br>±0.4	1.85<br>±0.04	23.1<br>±0.6	1.86<br>±0.03	24.2<br>±0.6	1.87<br>±0.07	22.7<br>±0.4	1.88<br>±0.07
dctP	23.5<br>±0.7	1.85<br>±0.04	23.7<br>±0.6	1.86<br>±0.06	18.8<br>±0.6	1.83<br>±0.05	21.8<br>±0.5	1.78<br>±0.05	22.3<br>±0.6	1.84<br>±0.06	21.3<br>±0.1	1.85<br>±0.05	21.3<br>±0.8	1.77<br>±0.04	21.2<br>±0.6	1.75<br>±0.06	21.1<br>±0.4	1.91<br>±0.06	19.7<br>±0.3	1.87<br>±0.03	19.9<br>±0.4	1.93<br>±0.06	19.9<br>±0.4	1.88<br>±0.06	18.9<br>±0.7	1.86<br>±0.04
dctM	27.6<br>±0.5	1.76<br>±0.07	27.7<br>±0.6	1.79<br>±0.06	23.1<br>±0.5	1.84<br>±0.06	25.9<br>±0.5	1.84<br>±0.05	26.2<br>±0.1	1.83<br>±0.06	25.8<br>±0.2	1.81<br>±0.05	25.5<br>±0.2	1.76<br>±0.07	25.3<br>±0.4	1.87<br>±0.04	24.9<br>±0.6	1.77<br>±0.06	23.9<br>±0.3	1.78<br>±0.05	24.5<br>±0.3	1.84<br>±0.05	24.7<br>±0.4	1.83<br>±0.07	23.5<br>±0.5	1.71<br>±0.04
dctA	25.5<br>±0.9	1.89<br>±0.05	26.5<br>±0.9	1.87<br>±0.03	20.9<br>±0.7	1.86<br>±0.06	23.9<br>±0.8	1.82<br>±0.06	24.0<br>±0.5	1.83<br>±0.07	23.0<br>±0.3	1.86<br>±0.06	23.1<br>±0.3	1.82<br>±0.05	22.9<br>±0.6	1.81<br>±0.07	23.7<br>±0.5	1.82<br>±0.05	23.2<br>±0.6	1.82<br>±0.06	22.8<br>±0.4	1.85<br>±0.05	22.9<br>±0.4	1.89<br>±0.04	22.0<br>±0.5	1.87<br>±0.03

[a] The lower value represents the standard deviation. The efficiencies were determined as described in the Methods section. The average efficiency per primer pair was used to calculate the ratios in Table S2.
